# Transcriptomic characterization of the enzymatic antioxidants FeSOD, MnSOD, APX and KatG in the dinoflagellate genus *Symbiodinium*

**DOI:** 10.1186/s12862-015-0326-0

**Published:** 2015-03-18

**Authors:** Thomas Krueger, Paul L Fisher, Susanne Becker, Stefanie Pontasch, Sophie Dove, Ove Hoegh-Guldberg, William Leggat, Simon K Davy

**Affiliations:** School of Biological Sciences, Victoria University of Wellington, Wellington, 6140 New Zealand; Laboratory for Biological Geochemistry, ENAC, École polytechnique fédérale de Lausanne (EPFL), Lausanne, 1015 Switzerland; School of Civil Engineering, University of Queensland, St Lucia, QLD 4072 Australia; School of Biological Sciences & ARC Centre of Excellence for Coral Reef Studies, University of Queensland, Brisbane, QLD, 4072 Australia; Global Change Institute, University of Queensland, Brisbane, QLD 4072 Australia; Comparative Genomics Centre, School of Pharmacy and Molecular Sciences & ARC Centre of Excellence for Coral Reef Studies, James Cook University, Townsville, QLD 4811 Australia

**Keywords:** Oxidative stress, ROS, Coral reefs, Catalase peroxidase, Superoxide dismutase, Ascorbate peroxidase, Antioxidant gene expression

## Abstract

**Background:**

The diversity of the symbiotic dinoflagellate *Symbiodinium* sp., as assessed by genetic markers, is well established. To what extent this diversity is reflected on the amino acid level of functional genes such as enzymatic antioxidants that play an important role in thermal stress tolerance of the coral-*Symbiodinium* symbiosis is, however, unknown. Here we present a predicted structural analysis and phylogenetic characterization of the enzymatic antioxidant repertoire of the genus *Symbiodinium*. We also report gene expression and enzymatic activity under short-term thermal stress in *Symbiodinium* of the B1 genotype.

**Results:**

Based on eight different ITS2 types, covering six clades, multiple protein isoforms for three of the four investigated antioxidants (ascorbate peroxidase [APX], catalase peroxidase [KatG], manganese superoxide dismutase [MnSOD]) are present in the genus *Symbiodinium*. Amino acid sequences of both SOD metalloforms (Fe/Mn), as well as KatG, exhibited a number of prokaryotic characteristics that were also supported by the protein phylogeny. In contrast to the bacterial form, KatG in *Symbiodinium* is characterized by extended functionally important loops and a shortened C-terminal domain. Intercladal sequence variations were found to be much higher in both peroxidases, compared to SODs. For APX, these variable residues involve binding sites for substrates and cofactors, and might therefore differentially affect the catalytic properties of this enzyme between clades. While expression of antioxidant genes was successfully measured in *Symbiodinium* B1, it was not possible to assess the link between gene expression and protein activity due to high variability in expression between replicates, and little response in their enzymatic activity over the three-day experimental period.

**Conclusions:**

The genus *Symbiodinium* has a diverse enzymatic antioxidant repertoire that has similarities to prokaryotes, potentially as a result of horizontal gene transfer or events of secondary endosymbiosis. Different degrees of sequence evolution between SODs and peroxidases might be the result of potential selective pressure on the conserved molecular function of SODs as the first line of defence. In contrast, genetic redundancy of hydrogen peroxide scavenging enzymes might permit the observed variations in peroxidase sequences. Our data and successful measurement of antioxidant gene expression in *Symbiodinium* will serve as basis for further studies of coral health.

**Electronic supplementary material:**

The online version of this article (doi:10.1186/s12862-015-0326-0) contains supplementary material, which is available to authorized users.

## Background

The photosynthetic dinoflagellate *Symbiodinium* sp. is a significant endosymbiont of a wide range of marine invertebrates and is a major contributor to the ecological success of reef-building corals in shallow tropical seas [1,2]. *In vitro* cultivation of coral symbionts allowed their identification as gymnoid dinoflagellates and led to their description as *Symbiodinium microadriaticum* in the middle of the 20^th^ century [3-5]. However, subsequent studies on cellular ultrastructure, cell morphology and physiological features suggested that *Symbiodinium microadriaticum* might in fact represent more than one species [6,7]. Through the application of molecular genetic tools, the genus *Symbiodinium* was revealed to contain a high degree of diversity, where genetic distances between *Symbiodinium* taxa are greater than between orders of non-symbiotic dinoflagellates [8-10]. Currently, nine major *Symbiodinium* clades (A-I), based on nuclear ribosomal DNA sequences and chloroplast 23S rDNA are established [11]. With the exception of clade E, multiple *Symbiodinium* types are recognized within each clade (e.g., A1, C3, F1), primarily based on sequence variations in the faster evolving internal transcribed spacer region 2 (ITS2) [11,12]. The now established phylogenetic relationship that designates main *Symbiodinium* clades and types within each clade is well supported by a number of different genetic markers [13-16]. Due to the multicopy nature of ITS2 and the resulting intragenomic variation, the application of an operational taxonomic unit (OTU) framework based on a statistical cluster-based approach with defined cut-offs, might be the closest approximation of a species definition in the genus *Symbiodinium* to date [17,18]. For important *Symbiodinium* clades, such as clade C that represents the major clade in Indo-Pacific corals, some authors have provided support that ITS2 types indeed represent evolutionarily distinct species by complementing the phylogenetic analysis with ecological, geographic and population genetic data [19,20].

The environmental sensitivity of the mutualistic association between *Symbiodinium* types and scleractinian corals has gained considerable attention over the last few decades through the phenomenon of coral bleaching. Bleaching results from a decline in endosymbiont density and/or photosynthetic pigment content in response to environmental stress, causing a paling of the coral [21], and can potentially lead to death of the coral colony. Large scale coral bleaching with subsequent mortality as a result of climate change-induced warming of the oceans represents a major threat to the functioning of coral reef ecosystems [22,23].

Variability in bleaching susceptibility and severity between different coral-*Symbiodinium* associations under similar temperature regimes has highlighted the role of *Symbiodinium* diversity in determining the performance of the coral “holobiont” (i.e., the entire community of living organisms that inhabit a coral colony [24-27]). While the physiological mechanisms of bleaching are not yet fully understood, the generation of reactive oxygen species (ROS) in the symbiont population under stress is thought to play an important role in the cellular bleaching cascade [28,29]. In this context, differences in ROS generation or antioxidant defences between different *Symbiodinium* types might contribute to the varying bleaching susceptibility between different coral species or even between populations of the same species.

Potentially damaging ROS, such as superoxide (O_2_^●-^) or hydrogen peroxide (H_2_O_2_), occur as side-products of photosynthesis and respiration [30], and antioxidants play an important role in preventing oxidative damage and, more generally, in maintaining redox homeostasis. The concerted response of the antioxidant network involves a number of pathways, and the scavenging of superoxide by superoxide dismutase (SOD) is considered the first line of defence. SOD is expressed in a number of metalloforms of which some are organelle-specific in higher plants (e.g., mitochondrial MnSOD or chloroplastic FeSOD; [31]). Downstream defences, such as peroxidases and catalases, deal with the end product of SOD activity, hydrogen peroxide. The activity of these peroxidases is linked to the availability of low molecular weight antioxidants, such as ascorbate and glutathione, which act as important cofactors and are essential to the H_2_O_2_ metabolism of photosynthetic organisms [32].

Changes in activity of SOD, ascorbate peroxidase (APX) and the bacterially-derived enzyme catalase peroxidase (KatG) have all been shown to be involved in the response to light and temperature stress in populations of both cultured and *in hospite Symbiodinium* [33-37]. Information on the protein structure and regulation of antioxidant gene expression, however, has been largely limited by a lack of nucleic acid sequence data, though the first steps towards this have been undertaken [38]. The application of Sanger sequencing and high-throughput sequencing technologies to the coral-dinoflagellate symbiosis has now provided a large body of transcriptomic data for a number of coral species [39-44], but only for a few *Symbiodinium* types [45-48]. The responses of a number of oxidative stress-targeted genes, such as ferritin, heat shock proteins, glutathione S-transferase, MnSOD and catalase have been documented in coral hosts exposed to environmental stress [44,49-52], though the mRNA expression of only one antioxidant gene (*apx1*) has been quantified in *Symbiodinium* (clade C only) [53,54]. Considering their pivotal role in coral biology, it is essential to broaden the transcriptomic toolkit to allow the assessment of antioxidant expression patterns in a variety of different *Symbiodinium* types.

The focus of this study was therefore to compile and characterize gene transcripts from four major enzymatic antioxidants (FeSOD, MnSOD, APX, and KatG) from different *Symbiodinium* clades. A more thorough analysis was done within clade C specifically, by looking at sequence similarities between ITS2 types. Degrees of sequence variation at the amino acid level, as well as phylogenetic relationships across different clades, were investigated. Furthermore, we demonstrated the utility of these data by measuring antioxidant gene expression and its corresponding enzyme activity in *Symbiodinium* B1 under short-term thermal stress.

## Results

### Transcriptomic characterization of antioxidants

A total of 87 antioxidant sequences, covering *Symbiodinium* clades A-F, revealed the presence of at least seven APX isoforms, four MnSOD isoforms, two KatG isoforms and one FeSOD form (Table 1).

**Table 1:** **Characteristics of isoforms of four enzymatic antioxidants in** ***Symbiodinium***

**Isoform**	**# of sequences**	**Identical sites**	**Clades represented**	**# of sequences with N-termini**	Subcellular location*	**TargetP reliability class [PLANT]**	**TargetP reliability class [NON-PLANT]**	**# of full length sequences**	**Ungapped length [aa]**	**Predicted molecular weight** ^**‡**^ **[kDa]**	**Predicted GPI-anchor [GPI-SOM/PredGPI]**
SymMnSOD1	6	89.1%	B, C	5	M/S^1^	M4-5; S3	M4-5; S3-5	4	263-265	28.6-28.9	No/No
SymMnSOD2	4	84.9%	B, D, F	4	M/S^2^	M5; S5	M3-5; S3	4	294-303	32.0-32.6	No/No
SymMnSOD3	5	55.5% (73.3%^3^)	A, B, D	3	S	S2-3	S1-2	2	246-247	26.5-27.2	No/No
SymMnSOD (others)	6		A, B	5	S	S2-3	S1-3	1	270	29.2	No/No
SymFeSOD	4	92.7%	A, B, E, F	1	C	-	-	1	201	21.9	No/No
SymAPX1	23	26.1%	A, B, C, D, F	14	C	-	-	6	391-397	42.4-43.6	No/No
SymAPX2	7	67.1%	A, B, C, D	2	M^4^/S^5^	M5; S1	M4; S1	2	311/362	33.9/38.6	No/No
SymAPX3	6	69.5%	A, D	5	C	-	-	4	317-320	34.7-34.9	No/No
SymAPX4	3	87.3%	B, D, F	1	C	-	-	1	308	33.6	No/No
SymAPX5	3	86.1%	A, D	2	C	-	-	2	453	49.0/49.1	No/No
SymAPX6	2	69.2%	A, B	1	M	M5	S5	1	328	36.0	Yes^7^/No
SymAPX (others)	2		A	2	?	Not recognized	Not recognized	0	-	-	
SymKatG1	12	63.6%	A, B, C, D, E, F	5	S	S2-5	S1-S5	1	486	54.4	Uncertain^7^/No
SymKatG2	4	73.1%	A, B, D	3	M^6^/S	M4; S5	S2-3	1	423	46.7	No/No

Molecular characterization of superoxide dismutase (FeSOD/MnSOD), ascorbate peroxidase (APX), and catalase peroxidase (KatG) protein isoforms identified in the genus *Symbiodinium*. Proportion of identical residues across represented clades is indicated for each isoform (for pairwise identities between types see Additional files 1, 5 and 7). Subcellular location indicated as cytosolic (C), mitochondrial (M), or as part of the secretory pathway (S). TargetP reliability classes (1-5) are indicated, where 1 indicates the strongest prediction. Length, molecular weights, and presence of GPI anchor are based on full-length sequences.

^*^based on results with “PLANT” settings in TargetP 1.1. for all sequences with complete N-terminal domains.

^‡^weights for SOD metalloforms and KatG refer to the monomer.

^1^only *Symbiodinium* C15 *M. digitata* SymMnSOD1.

^2^only *Symbiodinium* B1 Mf1.05b SymMnSOD2 rep_c13368 and *Symbiodinium* of clade D *A. hyacinthus* SymMnSOD2 [GenBank:GAFP01017905].

^3^including *Symbiodinium* A1 CCMP2467 SymMnSOD3 Assembly2 sequence.

^4^ *Symbiodinium* of clade D *A. hyacinthus* SymAPX2 [GenBank:GAFP01018157].

^5^ *Symbiodinium* of clade D *A. hyacinthus* SymAPX2 [GenBank:GAFP01007188].

^6^ *Symbiodinium* of clade D *A. hyacinthus* SymKatG2 [GenBank:GAFP01010883].

^7^GPI-SOM results based on C&N-terminal signal.

### Superoxide dismutases

**Figure 1:**
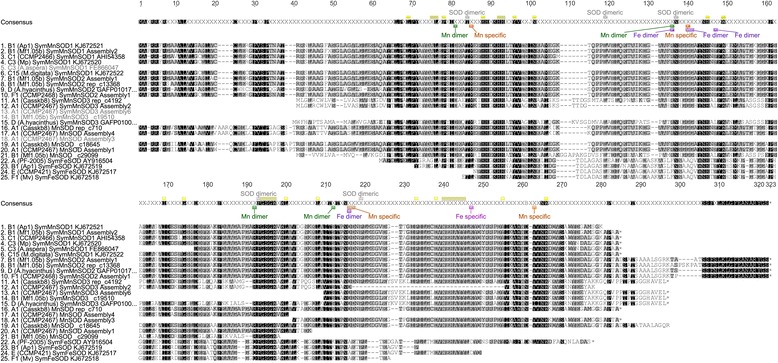
**Alignment of MnSOD (sequences 1–21) and FeSOD (22–25) protein isoforms from different** ***Symbiodinium*** **ITS2 types.** Residues used for identification of metalloform are highlighted on the consensus sequence. Conserved SOD residues (yellow), SOD dimer-specific Thr^84^, Leu^119^, Asn^137^, Phe^193^, and Pro^219^ (grey), MnSOD specific Met^85^, Gly^140^, Gln^216^, Asp^217^, Val^263^ (orange), FeSOD specific Asp^247^ (magenta), manganese dimer-specific Asp^81^, Arg^136^, Arg^192^, and Ser^212^ (green), and iron dimer-specific residues Phe^136^, Ala^140^, Gln^141^, Phe^147^, Ala^216^ (purple) have all been highlighted. Annotations were deduced from alignments with 17 characterized iron and manganese SOD sequences [55]. Ends of ORFs are indicated by asterisks. Shading indicates site-specific similarity over all sequences 100% (black), 80-100% (dark grey), 60-80% (light grey), and less than 60% (white), based on the Blosum62 score matrix with a threshold of 1. Sequence IDs contain ITS2 type, strain designation or source of isolation (in brackets) and GenBank accession number or contig/assembly designation (Additional file 11).

**Figure 2:**
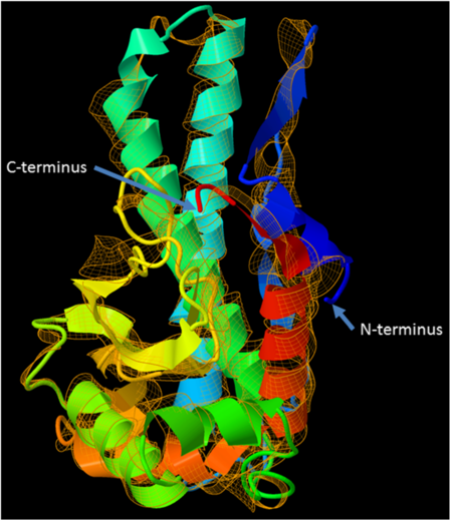
**Superimposition of SymMnSOD1 from** ***Symbiodinium*** **B1 and C1.** Shown are the predicted mature monomer structures of SymMnSOD1 from *Symbiodinium* B1 (Ap1, solid, C-score = 0.30) and *Symbiodinium* C1 (CCMP2466, orange mesh ribbon, C-score = 0.32). Colour temperature indicates direction from the N- to the C-terminus (blue to red).

Both, MnSOD and FeSOD represent dimeric forms that were highly conserved across clades. Transcripts of *fesod* were only successfully amplified from *Symbiodinium* B1, E and F1, while none were found in any of the available EST libraries. Obtained partial sequences of *Symbiodinium* FeSOD (SymFeSOD) covered 66-74% of the encoded total protein length of 201 aa (21.9 kDa), as determined from a previously published *Symbiodinium* clade A sequence (PF-2005; [GenBank:AY916504]) (Figure 1). Encoded protein sequence length within each MnSOD isoform was similar across clades (within 1–9 aa of each other), but absolute length between isoforms varied by up to 19% (e.g., SymMnSOD3 vs. SymMnSOD2). Mean pairwise protein identities across clades were highest for SymFeSOD (96% ± 2%; mean ± SD), SymMnSOD1 (94% ± 2%), and SymMnSOD2 (90% ± 3%), with near identical amino acid sequences (96-97%) for MnSOD1 within clade C (C1, C3, C15) (Figure 1, Additional file 1). The predicted tertiary monomer structure of SymMnSOD1 from *Symbiodinium* B1 and C1 was also highly similar (Figure 2). I-tasser modelling returned bacterial SODs as the top five structural Protein Data Bank (PDB) analogues for both types. Pairwise identities between clades were lower for SymMnSOD3 (73% A1 vs. D) and other MnSOD (41%) sequences, partially due to limited sequence data for the latter ones (Figure 1, Additional file 1). The lowest pairwise identities between full-length sequences were usually encountered between *Symbiodinium* of clade D and all other clades (e.g., 87-89% D vs. B1 and F1 for MnSOD2; 73% D vs. A1 for MnSOD3).

Dinoflagellate spliced leader sequences were located 45–53 bp upstream of the start codons of SymMnSODs. Signal peptides, but no transmembrane domains were found in all N-terminal domains (Additional file 2). However, prediction strength of their subcellular locations given by TargetP 1.1 was low for SymMnSOD1 and 2 (reliability class 3–5), assigning them to the mitochondrial or secretory pathway (Table 1). The remaining MnSOD isoforms were identified as part of the secretory pathway. Variants of the prominent ancient plastid-targeting “FVAP”-type motif (“FVSP” in MnSOD1 + 2) were found in the transit peptide region of almost all sequences (Additional file 2). No signal peptide was found for SymFeSOD.

### Ascorbate peroxidase

A total of 46 *apx* sequences were obtained from PCR amplifications and EST library entries, and they were grouped according to their amino acid sequence characteristics (SymAPX1-SymAPX6) as long as members of these six groups were found in at least two *Symbiodinium* clades (Table 1). SymAPX1 and 2 were identified as hybrid ascorbate-cytochrome *c* peroxidases (APX-CcP), whereas all others matched common ascorbate peroxidase sequences (Additional file 3). N-terminal signal peptides were only found in SymAPX2 and 6, and two unclassified sequences, assigning SymAPX2 and 6 to either secretory or mitochondrial pathways (Table 1, Additional file 4). TargetP did not recognize the Phobius-predicted signal peptide in the two unclassified sequences. All other APX sequences appear to be cytosolic. Overall protein length varied considerably between cytosolic isoforms (308–453 aa), and multiple start codons could be found within the same ORF. Nevertheless, ATGpr software’s prediction of the “true” start codon in SymAPX1 was supported by the location of the spliced leader, found 58 bp upstream of the coding sequence in the SymAPX1 isoform of *Symbiodinium* F1 (Mv). This was, however, the only APX sequence where the spliced leader primer, in conjunction with a reverse primer, successfully amplified a gene fragment.

**Figure 3:**
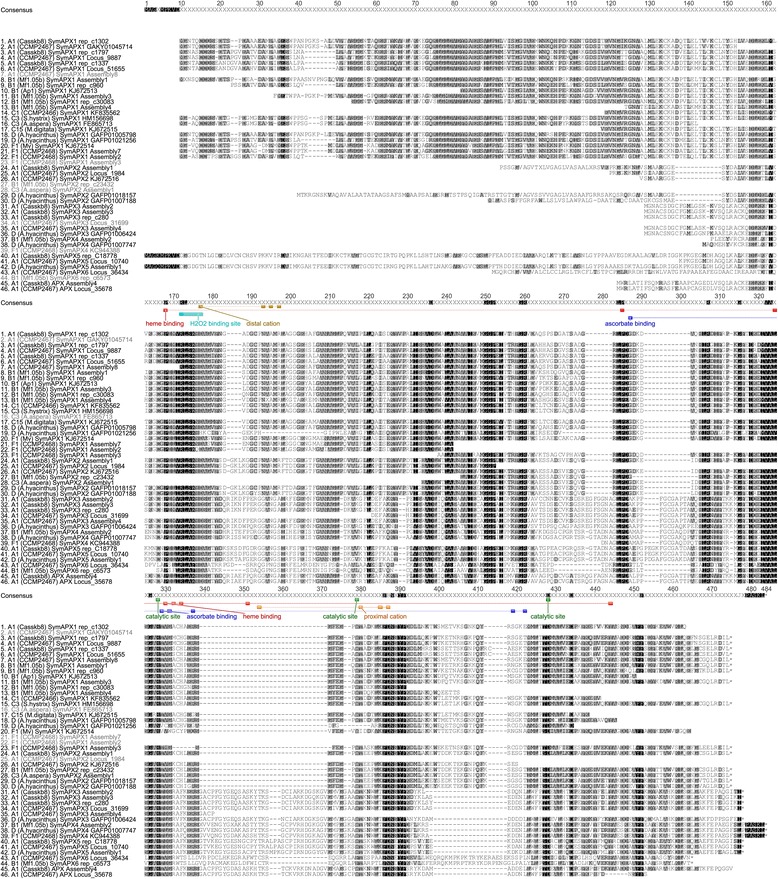
**APX protein alignment for different** ***Symbiodinium*** **ITS2 types.** Annotations indicate functionally important residues involved in binding of heme (red), ascorbate (blue) and cations (proximal [orange] and distal [ochre]). Binding sites for hydrogen peroxide (cyan) and residues involved in the formation of the catalytic site (green) are highlighted. Annotations were deduced from alignments with characterized chlorophyte, bryophyte, lycophyte, and higher plant sequences [56]. Ends of ORFs are indicated by asterisks. Sequence IDs consist of ITS2 type, strain designation or source of isolation (in brackets), APX isoform and GenBank accession number or contig/assembly designation (Additional file 11). Shading for sequence similarity is identical to that of Figure 1.

**Figure 4:**
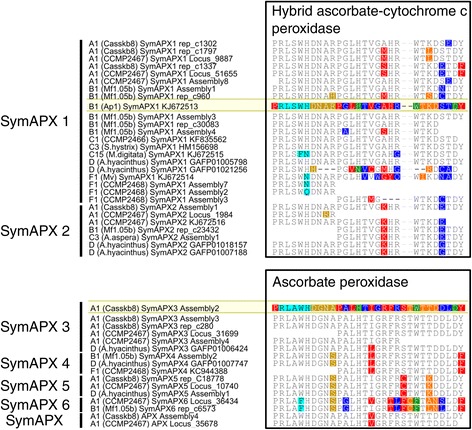
**Variation of functional amino acid residues between** ***Symbiodinium*** **types and APX isoforms.** Shown is a sequential alignment of residues involved in binding of heme (red), ascorbate (blue), hydrogen peroxide (cyan), and cations (proximal [orange] and distal [ochre]), as well as residues involved in the formation of the catalytic site (green) (*sensu* Figure 3). Differences are highlighted in relation to reference sequences (yellow) for hybrid ascorbate-cytochrome *c* peroxidases (SymAPX1 + 2) and typical ascorbate peroxidases.

Length differences between APX isoforms (relative to SymAPX1) were driven by a number of deletions in the N-terminal domain (SymAPX2-6) and between residues 397–418 (SymAPX3-5), but also by large insertions around residues 275–302 (SymAPX3-5) and 339–371 (SymAPX3-6) (Figure 3). Pairwise amino acid identities were considerably lower than for SODs, but varied depending on isoform. Full-length comparison of SymAPX1 showed pairwise identities of 63-64% (A1 vs. B1), 64-67% (A1 vs. C3) and 76% (B1 vs. C3), or even lower values (e.g., 56-62% for A1 vs. F1 [Additional file 5]). In comparison, the similarities of SymAPX3 between A1 and D were 73-74%. Variation in SymAPX1 within clade C was predominantly found in C15 (91% pairwise identity to C1 and C3), whereas C1 and C3 were nearly identical (99.6-100%). Functionally important residues involved in binding of heme and hydrogen peroxide, as well as the formation of the catalytic site, were largely conserved within each APX isoform, with the exceptions of *Symbiodinium* C15 and F1, where Trp^175^ and His^176^ in the hydrogen peroxide binding site of SymAPX1 were replaced by Phe^175^ in C15 and Asn^176^ in both (Figures 3 and 4). In *Symbiodinium* F1 (Mv), five of the seven ascorbate binding site residues and two of the six heme binding site residues differed from those of other clades.

### Catalase peroxidase

**Figure 5:**
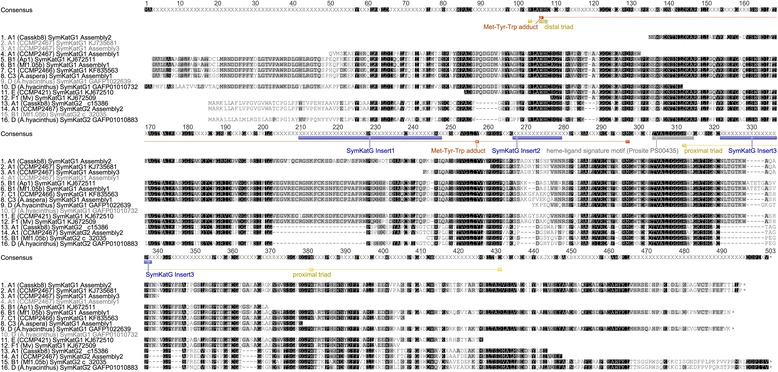
**Alignment of KatG protein sequences for different** ***Symbiodinium*** **ITS2 types.** Highlighted are KatG-specific features and the location of SymKatG inserts (blue). Annotations were deduced from alignments of proteins from *Haloarcula marismortui* (RCS PDB 1ITK_A) and *Burkholderia pseudomallei* (MWV_A). Ends of ORFs are indicated by asterisks. Sequence IDs consist of ITS2 type, strain designation or source of isolation (in brackets), KatG isoform and GenBank accession number or contig/assembly designation (Additional file 11). Shading for sequence similarities is identical to that of Figure 1.

The two KatG isoforms found in *Symbiodinium* differed mainly by two insertions in SymKatG1 between the consensus residues 88–95, 202–228 and 321–334 (Figure 5). The proximal heme-ligand signature motif (TVALIGGGHTL; Prosite PS00435) differed slightly between both isoforms, but was highly conserved in each one. Signal peptides for the secretory (SymKatG1 and SymKatG2) and/or mitochondrial pathway (D [*Acropora hyacinthus*] SymKatG2 [GenBank:GAFP01010883]) were found (Table 1, Additional file 6). TargetP reliability of the assigned pathway was, however, highly variable between sequences. Mean pairwise identities across clades was 80% ± 9% for SymKatG1 (clades A-F) and 77% ± 2% for SymKatG2 (clades A, B, D), respectively. *Symbiodinium* C1 and C3 shared the highest pairwise identity (98.1% for SymKatG1; Additional file 7).

**Figure 6:**
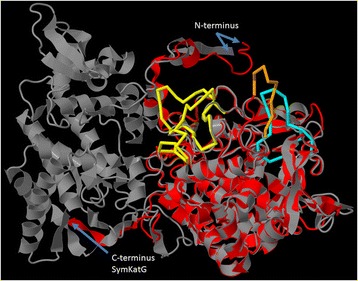
**Superimposition of bacterial and** ***Symbiodinium*** **KatG.** Shown are the predicted mature SymKatG1 monomers from *Symbiodinium* B1 (Mf1.05b, C-score = 0.09, red) superimposed with the crystal structure monomer from *Haloarcula marismortui* (RCS PDB ID 1ITK_A, grey). SymKatG inserts 1 (yellow), 2 (orange) and 3 (cyan) have been highlighted in the *Symbiodinium* protein (cf. Figure 5, Additional file 8).

Comparison with bacterial KatGs revealed that SymKatGs are, in general, shorter and cover only the N-terminal domain of the bacterial form. Residues of the distal (Trp, Arg, His) and proximal triad (His, Asp, Trp), as well as the Met-Tyr-Trp adduct are, however, all conserved. Structural modelling of the SymKatG from *Symbiodinium* B1 (Mf1.05b) identified the crystal structure of the bacterial catalase-peroxidase from *Haloarcula marismortui* (PDB:1itk) as the best structural analog (TM-score = 0.839). In comparison to bacterial KatGs, SymKatGs contain up to three sequence extensions (SymKatG inserts 1–3; Figure 5, Additional file 8) at positions 210–247 (only SymKatG1; numbering according to Figure 5), 267–279 (both isoforms) and 322–338 (SymKatG1). Superimposition of the SymKatG from B1 (Mf1.05b) with *Haloarcula marismortui* [PDB:1itk] indicates that these inserts form large loops, primarily on the surface of the protein (Figure 6). Inserts 2 and 3 were also the main source of sequence variability between *Symbiodinium* types, with multiple amino acid inserts or deletions (Figure 5).

### Protein phylogeny

**Figure 7:**
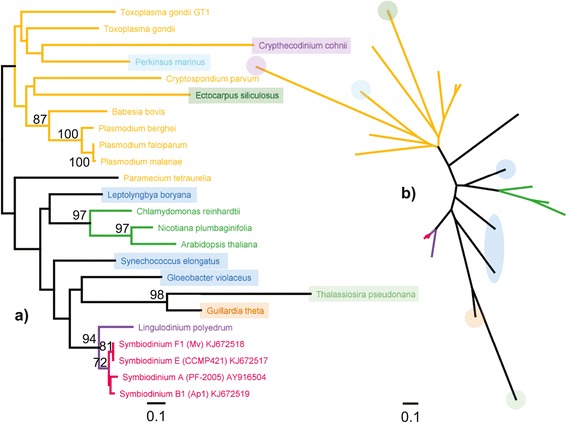
**FeSOD protein phylogeny.** Phylogeny of *Symbiodinium* FeSOD sequences and those of other taxa, inferred from ML analysis (WAG + I + G model, 139 aa alignment length); rooted **(a)** and unrooted **(b)** trees. Scale bars indicate the number of amino acid substitutions, and bootstrap percentages >70% are indicated on nodes. The locations of specific sequences in the unrooted tree are indicated by coloured circles. Colouring of branches refers to lineages containing *Symbiodinium* (red), dinoflagellates (purple), chlorophytes and higher plants (bright green), and other alveolates (orange, including the heterokont *Ectocarpus siliculosus*).

*Symbiodinium* FeSOD sequences were most similar to those of the dinoflagellate *Lingulodinium polyedrum*. The FeSOD proteins from both of these dinoflagellates were, however, more closely related to cyanobacterial sequences (based on patristic distance) than to the general alveolate cluster; the latter also included the dinoflagellate sequence from *Crypthecodinium cohnii* (Figure 7). Cryptophyte and bacilliariophycean sequences formed a separate branch outside of the alveolate cluster, with another cyanobacterial sequence at its root. Overall tree robustness was low for both SOD metalloforms, with strong node support only present in lower branches.

**Figure 8:**
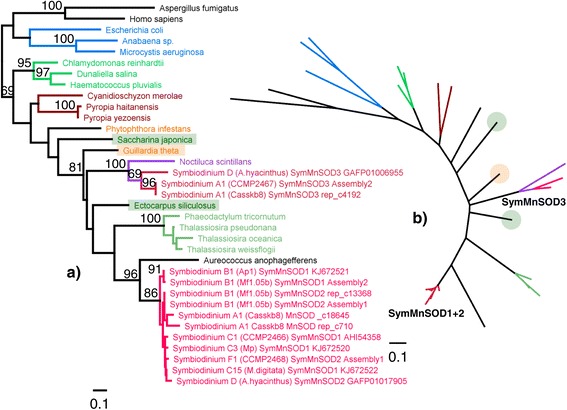
**MnSOD protein phylogeny.** Protein phylogeny as rooted **(a)** and unrooted **(b)** trees based on ML analysis (WAG + I + G model, 230 aa alignment length). Colouring of branches refers to lineages containing *Symbiodinium* (red), dinoflagellates (purple), bacillariophyceans (moss green), bacteria (blue), rhodophytes (burgundy), and chlorophytes and higher plants (bright green).

SymMnSOD1 and 2 were grouped into a single cluster, with the pelagophyte *Aureococcus anophagefferens* as closest relative. Within this *Symbiodinium* cluster, only SymMnSOD1 from B1 were identified as a basal group, while all other sequences lacked any cladal resolution. SymMnSOD3 was most closely related to another dinoflagellate, *Noctiluca scintillans*, grouping it together with *Ectocarpus siliculosus* as well as the bacillariophycean cluster (Figure 8).

**Figure 9:**
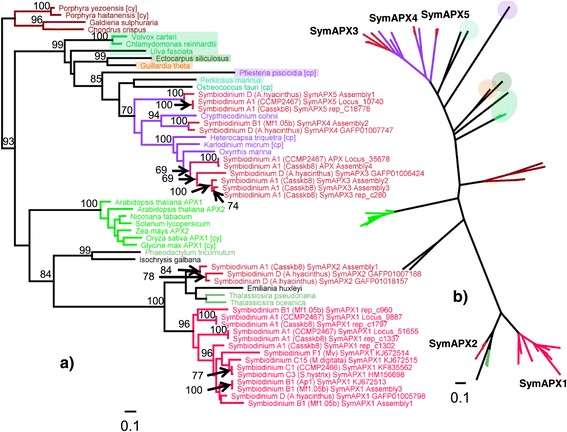
**APX protein phylogeny.** Protein phylogeny as rooted **(a)** and unrooted **(b)** trees based on ML analysis (LG + I + G + F model, 321 aa alignment length) using rhodophytes as the outgroup. Colouring of branches refers to lineages containing *Symbiodinium* (red), dinoflagellates (purple), bacillariophyceans (moss green), chlorophytes (green), and higher plants (bright green).

*Symbiodinium* APX sequences expressed a strong dichotomy relative to the rhodophyte outgroup. The tip of the lower branch (Figure 9b) separated the two clusters of SymAPX1 and SymAPX2, with the latter also containing sequences from *Thalassiosira* sp. (Bacillariophyceae) and *Emiliania huxleyi* (Prymnesiophyceae). The SymAPX1 cluster expressed some degree of cladal separation, with sequences from clades A and B usually located basally to more derived ITS2 types. SymAPX3-5, including unclassified SymAPX, formed well-separated clusters at the tip of the upper branch that comprised all dinoflagellate sequences used in the analysis. With a chloroplastic *Pfiesteria piscicida* sequence at its base, the dinoflagellate branch also enclosed two APX sequences from *Perkinsus marinus* and *Ostreococcus tauri* (Figure 9). Node support for cladal separation (A1 or B1 vs. D) within each SymAPX isoform was very high (100%).

**Figure 10:**
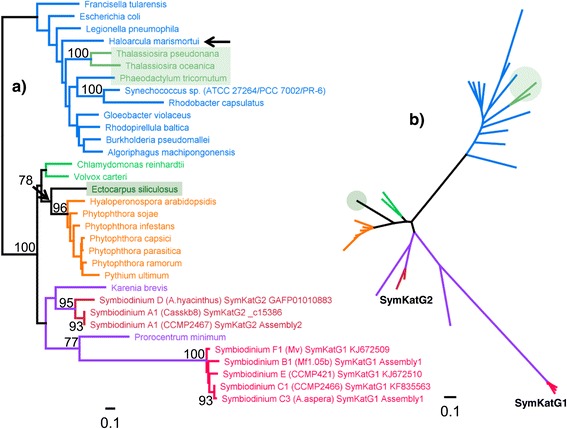
**KatG protein phylogeny.** Protein phylogeny as rooted **(a)** and unrooted **(b)** trees based on ML analysis (WAG + I + G model, 328 aa alignment length). Colouring of branches refers to lineages containing *Symbiodinium* (red), dinoflagellates (purple), bacteria (blue), bacillariophyceans (moss green), chlorophytes (green), and heterokont oomycetes (orange).

Relative to the original bacterial cluster, which also contained bacilliariophycean sequences, dinoflagellate KatGs form a strongly supported sister branch to a branch that contains chlorophyte, oomycete and phaeophycean sequences (Figure 10). While SymKatG1 and 2 form separate branches within the dinoflagellate cluster, the phylogenetic distance relative to other dinoflagellates is especially high for SymKatG1. In fact, the patristic distance between SymKatG2 (A1) and SymKatG1 (F1) is about the same as the distance between SymKatG2 (A1) and the cyanobacterium *Synechococcus* sp. (2.024 vs. 1.991). Cladal separation was evident for both isoforms. Resolution for SymKatG1 was limited though, indicating only a basal *Symbiodinium* F1 sequence, relative to a cluster of sequences from clades B, C, and E.

### Protein activity and gene expression responses of enzymatic antioxidants in *Symbiodinium* B1 under short-term thermal stress

Exposure to 33°C led to an arrest of cell population growth, relative to the control (μ = 0.13 ± 0.04 d^−1^ at 40–50 μmol quanta m^−2^ s^−1^; mean ± SE, N = 6), and a 16% decline in F_v_/F_m_ (Table 2). This was, however, not accompanied by a decline in chlorophyll *a* (Chl *a*) content. While no significant effect of temperature on Chl *a* or protein content was found over time, protein content per cell on Day 3 tended to be higher at 33°C (p = 0.0733), so we normalized enzymatic activity to cell number rather than protein content. A significant temperature effect on antioxidant enzyme activity was only found for APX, whose activity at 33°C was approximately 30% higher after three days (Table 2).

**Table 2:** **Effect of temperature on physiological variables of** ***Symbiodinium*** **B1**

**Response variable**	**°C**	**Day 0**	**Day 1**	**Day 3**	***F***_**time x temperature**_
Cell density	25	148920 ± 6142	161651 ± 6553	216050 ± 10864	*F*_2,9_ = 40.3486,
	33	151759 ± 11152	128704 ± 6988	126389 ± 5798	p < 0.0001*
F_v_/F_m_	25	0.51 ± 0.01	0.51 ± 0.01	0.51 ± 0.01	*F*_2,9_ = 19.5472,
	33	0.51 ± 0.02	0.46 ± 0.02	0.43 ± 0.01	p = 0.0005*
Chl *a*	25	0.85 ± 0.05	0.89 ± 0.03	0.77 ± 0.05	*F*_1.263,12.63_ = 0.6068,
[pg cell^−1^]	33	0.90 ± 0.03	1.00 ± 0.06	0.92 ± 0.08	p = 0.4875
Protein	25	3.82 ± 0.31	4.62 ± 0.40	4.67 ± 0.28	*F*_2,9_ = 2.9263,
[pg cell^−1^]	33	4.74 ± 0.46	4.23 ± 0.38	5.57 ± 0.35	p = 0.1049
SOD	25	389.6 ± 25.7	453.8 ± 48.8	541.0 ± 33.4	*F*_2,9_ = 1.7356,
[nU cell^−1^]	33	486.3 ± 44.4	437.9 ± 37.9	594.1 ± 34.3	p = 0.2304
APX	25	2.01 ± 0.22	2.23 ± 0.26	2.31 ± 0.22	*F*_2,9_ = 4.2666,
[nU cell^−1^]	33	2.17 ± 0.24	2.14 ± 0.25	2.99 ± 0.29	p = 0.0497*
KatG	25	17.43 ± 3.69	15.11 ± 2.93	13.77 ± 1.95	*F*_2,9_ = 0.7105,
[nU cell^−1^]	33	19.86 ± 2.58	20.74 ± 3.34	19.45 ± 2.30	p = 0.5170

Temperature effects on viability and physiological variables in *Symbiodinium* B1 (in culture) over three days of exposure to 25°C or 33°C. Values represent mean ± SE (N = 6). Significant rmANOVA effects are indicated by asterisks.

**Figure 11:**
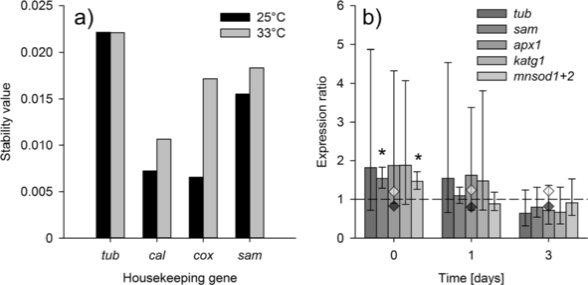
***Symbiodinium*** **B1 gene expression. (a)** Expression stability of housekeeping genes over three days exposure to 25°C and 33°C in *Symbiodinium* B1 (N = 6). **(b)** Gene expression of ß-tubulin (*tub*), S-adenosyl methionine synthetase (*sam*), ascorbate peroxidase (*apx1*), catalase peroxidase (*katg1*) and manganese superoxide dismutase (*mnsod1 + 2*) in B1 at 33°C relative to controls. Numbering of GOI designations refer to isoform numbering presented in this study (cf. Table 1). Values are means ± SE (N = 6). Expression of the HKG calmodulin (white diamonds) and cytochrome *c* oxidase subunit 1 (black diamonds) is indicated. Asterisks indicate significant up-regulation relative to the control (p < 0.05).

*Cox* and *Cal* were identified as the best combination of HKGs, with a combined stability value of 0.006 and 0.008 at 25°C and 33°C, respectively (Figure 11a). Relative expression of the remaining house-keeping genes and the target genes at 33°C showed no significant change over time, apart from the observation that *sam* and *mnsod* were expressed to a significantly stronger degree at 33°C relative to 25°C on Day 0 (Figure 11b). The high variability in relative expression for most of the genes on Days 0 and 1 had disappeared by Day 3, where all replicates had a similar range of expressions. These were, however, not significantly different from gene expression values in the control treatment.

## Discussion

The large diversity in the dinoflagellate genus *Symbiodinium* has been inferred from the extensive variation in the rDNA ITS2 region [9]. While different genetic markers have been studied in the context of taxonomic resolution and phylogenetic links within the genus, individual gene phylogenies might not always be evolutionary identical (reviewed in [57]). However, to what degree this genetic diversity is reflected on the amino acid level of functionally important genes, such as antioxidant genes, has never been investigated. This study provides, for the first time, an overview of the intrageneric diversity of the antioxidant repertoire in the genus *Symbiodinium*. Protein sequence characteristics and phylogenetic analyses indicated a high degree of sequence conservation at the amino acid level for SOD isoforms, but considerable variation in peroxidases. Gene expression and protein activity of MnSOD, APX and KatG were successfully measured in *Symbiodinium* B1 at different temperatures. Significant changes in protein activity at high temperatures were, however, limited to APX and not associated with increases in expression of the underlying gene.

### SOD isoforms are highly conserved between *Symbiodinium* types

Structurally, all *Symbiodinium* MnSODs and FeSODs found here are dimeric, a characteristic that is generally found in prokaryotes rather than eukaryotes, in which MnSODs are usually tetrameric [30]. At least three MnSOD isoforms exist in *Symbiodinium*, agreeing with the electrophoretic detection of three to four MnSOD isoforms in *Symbiodinium* from two different host species [58]. The monomeric weights for the SymMnSODs (28–33 kDa) and SymFeSOD (21.9 kDa) correspond roughly to previously reported native (dimeric) weights of 43.5 kDa (MnSOD) and 49.5 kDa (FeSOD) [59]. Interestingly, no FeSOD sequence was found in any of the EST libraries queried and it was not possible to readily amplify and sequence mRNA from some of the *Symbiodinium* types. This could be related to the fact that *fesod* seems to be expressed at a very low level, as high C_t_-values and problems with non-specific amplification thwarted attempts to reliably quantify expression of this gene in *Symbiodinium* B1. The difficulties encountered in amplifying *fesod* prevent conclusions being made as to the true diversity of this form, though at least two protein isoforms were reported for *Symbiodinium* of the sea anemone *Anemonia viridis* [58]. While previous studies have established the proteomic presence of all three SOD isoforms (CuZnSOD, MnSOD, and FeSOD) in *Symbiodinium* [58,59], the random primer approach used here detected only the manganese and iron SOD forms.

Sequence homology between *Symbiodinium* types was very high for both MnSOD and FeSOD. As a result, these enzymes seem to be structurally almost identical between *Symbiodinium* types, suggesting similar catalytic properties. This homology is also reflected in the fact that it was not possible to phylogenetically distinguish any of the seven ITS2 types within the SymMnSOD1 + 2 cluster. The high degree of SOD amino acid conservation, despite the genetic radiation of the genus, shows the limitations of genetic variation in this functional gene, as mutation to critical residues involved in structural characteristics and catalytic functions might be lethal, given the pivotal role of SOD as the first line of antioxidant defence [60]. Although the dimeric nature of both SOD metalloforms indicates a bacterial origin, the protein phylogeny reflects a close relationship to cyanobacterial sequences only for FeSOD. All of the MnSOD isoforms, on the other hand, have evolved within the stramenopile cluster, consistent with a red-algal origin. Interestingly, a MnSOD from the hacrobian cryptophyte *Guillardia theta* (a Stramenopile-Alveolata-Rhizaria [SAR] sister lineage) was also found within this cluster.

The suggested secretory nature of 14 of the 17 SymMnSOD sequences with a complete N-terminal domain requires further investigation with regard to the subcellular locations of these forms due to the low reliability of the prediction strength using the “plant” setting in TargetP 1.1 and the peculiar nature of transit peptides in dinoflagellates [61]. Interestingly, a variant of the plastid-targeting “FVAP”-type motif was found in almost all sequences and it should be noted that despite the predicted mitochondrial location, SymMnSOD1 and 2 fulfilled two of the three proposed cut-off criteria for transit peptides of potential plastid-targeted proteins (FVAP-type motif and less than 7.7% acidic residues [61]). With mitochondrial and chloroplast signal peptides having a number of common sequence features [62], further localisation studies beyond the bioinformatical level applied here, are required. Similarly, the absence or low reliability of a chloroplastic target signal in photosynthetic and heterotrophic dinoflagellate FeSODs from *Symbiodinium* of clade A (PF-2005, [GenBank:AY916504]), *Crypthecodinium cohnii* [GenBank:ABQ23892] and *Lingulodinium polyedrum* [GenBank:AF289824] emphasizes the need for such investigations, but also raises the question as to what extent plastid targeting signals differ in dinoflagellates, potentially assigning them to multiple compartments [63], or whether these FeSODs represent cytosolic forms, as suggested for heterotrophic dinoflagellates [64].

### Extended loops and a shortened C-terminal domain characterize *Symbiodinium* catalase peroxidases

Catalase peroxidases (KatG) are bifunctional heme peroxidases which, in contrast to other peroxidases, display both catalase and peroxidase activity. These enzymes are of bacterial origin and resulted from a gene duplication event in which both protein domains remained fused while the C-terminal domain lost its catalytic activity [65]. While the exact function of this domain is unknown, it is suspected to stabilize the protein and its absence affects the spatial layout of the heme-binding residues in the catalytic domain [66,67]. The reduction or absence of the C-terminal domain has been previously observed in other dinoflagellates [68]. Evolutionarily, dinoflagellate KatGs belong to the minor KatG clades, which are intermediate between bifunctional KatGs and uni-functional peroxidases like APX (including hybrid APX-CcP) and cytochrome *c* peroxidases [69]. The observed SymKatG Inserts 1 and 3 are essentially an extension of the characteristic first and second KatG loop [70], while SymKatG Insert 2 is a unique feature only shared by *Prorocentrum minutum*. The observed sequence variability between *Symbiodinium* types in these loops might be of evolutionary importance, since Loops 1 and 2 are structurally relevant for the access of hydrogen peroxide to the prosthetic heme group and thus catalytic activity [71,72]. Structural modifications, with alteration of amino acid residues involved in substrate and cofactor binding, can alter catalytic rates and activity of these enzymes [73], and might therefore be under selective pressure. Further research is clearly needed to bridge the gap between sequence variations and their functional consequences in different *Symbiodinium* types. Purifying and characterizing these enzymes biochemically will allow further investigations on the thermal stability and substrate affinity (K_m_-values) of these enzymes.

### Sequence evolution of peroxidases reflect genetic radiation of the genus *Symbiodinium*

Isoforms of APX and KatG expressed a greater number of amino acid substitutions between *Symbiodinium* types than did SOD metalloforms, which indicates a higher rate of sequence evolution for these two phylogenetically-related heme peroxidases. APX and KatG phylogenies distinguished separate *Symbiodinium* clades within their respective isoform clusters, even to the point of resolving subcladal diversity between C1, C3 (the ancestral lineage of the C clade), and C15. Given that clade A sequences tended to be basal to most clusters, it is conceivable that these two enzymes have co-evolved with the genetic radiation of the genus. Further data are, however, required to validate this. Sequence divergence of both heme peroxidases within the genus *Symbiodinium* is nevertheless large (with full length identities as low as 61% for SymAPX1 from types A1 and B1), and is much higher than the protein divergence seen at the genus level of higher plants (cf. Figure 9). However, while consistent features of cytosolic APXs of higher plants were found in SymAPX1 + 2 (characteristic His^334^ [corresponding to H^169^ in the cytosolic APX of pea [74]] and Phe^340^ [Phe^175^ in the cytosolic *Arabidopsis* APX [75]], no typical chloroplastic isoform could be identified.

The high degree of sequence divergence between peroxidase isoforms, as well as intercladal sequence variability within each isoform, is in contrast to the high conservation in both SOD metalloforms. This lower sequence evolution in *Symbiodinium* SODs might be due to either lower mutation rates or a higher selective pressure on their conserved molecular function as a first line of defence. On the other hand, genetic redundancy (multiple genes with the same proteomic function potentially compensating for modification or inactivation of one of them [76]) in downstream defence lines, such as the removal of hydrogen peroxide, could provide ‘evolutionary space’ for alterations in peroxidase sequences to occur. This antioxidant redundancy, demonstrated in mutants of higher plants [77], has not yet been shown for *Symbiodinium*, partially due to the lack of available sequences for most of these genes. However, on an evolutionary scale, this redundancy might have provided the opportunity for the development of the observed sequence alterations if they become evolutionarily relevant in providing the means to more effectively detoxify ROS. Variability in downstream antioxidant responses, related to the removal of hydrogen peroxide, has already been shown to be a key difference between different *Symbiodinium* types under thermal stress at the protein level [36].

The presence of multiple, nuclear-encoded class I peroxidases (KatG, APX, APX-CcP) in the transcriptome of *Symbiodinium*, with separate modes of sequence evolution for specific isoforms, suggests that these were acquired by endosymbiosis or horizontal gene transfer (HGT). For example, APX isoforms SymAPX3-5, located within the alveolate cluster, are in stark contrast to SymAPX1-2, which were closely related to proteins from bacillariophyceae and haptophytes. *Symbiodinium* belongs to a cluster of dinoflagellates, called the Gymnodiniales-Peridiniales-Prorocentrales (GPP) complex, which contains a number of genera, whose genes were derived from plastid acquisition from bacillariophyceae and haptophytes [78], as well as through secondary and tertiary endosymbiosis (e.g., *Karlodinium venificum*) [79]. Our results add to other findings that *Symbiodinium* is no exception with regard to “foreign” genes [46,80,81]. For KatGs, a transfer of ancestral bacterial KatG genes into marine eukaryote genomes through horizontal gene transfer has been suggested; specifically, KatG acquisition from cyanobacteria or marine bacteria in diatoms has been proposed [67]. SymKatGs demonstrate, however, a more reduced C-terminal region in comparison with diatoms. The suggested common KatG ancestry of dinoflagellates with evolutionarily distant stramenopile and chlorophyte proteins (all of them sharing the reduced C-terminal region) could suggest multiple independent gene transfer events for KatGs.

### Antioxidant gene expression in *Symbiodinium* B1

In order to normalize antioxidant expression, a number of potential HKGs were chosen that had previously been shown to be suitable for thermal experiments with *Symbiodinium* C3 [82]. The Normfinder analysis for expression stability agreed with this previous study, in that *tub* is the least stable HKG candidate for studying thermal stress. However, expression of *cal* and *cox* was more stable in *Symbiodinium* B1 than *sam*, in contrast to the findings for C3. Large variation in gene expression between replicates was evident in this study and, though natural variation in gene expression has been shown for *in hospite Symbiodinium* populations *in situ* [83], there is no obvious explanation as to why this occurred under the controlled lab settings used here. Changes in gene expression in *Symbiodinium* under stress have consistently been found to be small [83-86]; thus, minimizing variation between replicates is crucial to resolve small, but potentially biologically meaningful changes in gene expression.

Clearly, the thermal scenario tested here arrested population growth and moderately lowered the maximum quantum yield of photosystem II in *Symbiodinium* B1. It was, however, insufficient for evoking a significant response in the expression and activity of most of the antioxidants monitored. Indeed, only cellular hydrogen peroxide scavenging through APX activity was significantly elevated after three days at 33°C in this *Symbiodinium* type. This lack of response within the timeframe tested, and the high degree of variation in gene expression between replicates, made it impossible to correlate changes in antioxidant gene transcripts and enzymatic activity in *Symbiodinium* B1. Further research about the level of regulation of these important antioxidant genes in *Symbiodinium* under stress is needed, as some studies highlight the importance of post-translational regulation in dinoflagellates [87-89]. The sequence data compiled here, and their successful application to measurements of antioxidant gene expression in *Symbiodinium* B1, provides the foundation for such studies.

## Conclusions

The physiological traits that are associated with particular *Symbiodinium* genotypes have profound implications for their hosts, especially in obligate associations such as the coral-*Symbiodinium* symbiosis. The use of a consistent genetic classification of *Symbiodinium* types provides the foundation for any systematic investigation of the link between genetic identity and ecophysiology. However, genetic differences, as assessed by fast-evolving markers such as ITS2, should not *a priori* lead to the assumption of fundamentally different physiologies. Differences in “machinery” (interplay of cellular pathways to maintain cellular homeostasis) between types has been well studied, but to what extent differences in “hardware” (protein structures and catalytic properties) contribute to the particular physiology of different *Symbiodinium* types should also be considered. This study shows that superoxide dismutase enzymes are structurally highly similar across the investigated clades and types, whereas hydrogen peroxide-scavenging peroxidases display considerable variation in predicted amino acid residues. Intriguingly, these findings correlate to some extent with the observed similarity in SOD baseline activities between different types, but higher variability in downstream antioxidant enzymes under the same environmental settings [36]. Considering the role of oxidative stress in coral bleaching, and the connection between differential bleaching susceptibility and symbiont diversity, the presented dataset provides an important tool for further comparative studies related to the functioning and regulation of antioxidant genes in *Symbiodinium*.

## Methods

### Sequence characterization and phylogenetic analysis of antioxidant genes

#### Symbiodinium *types, RNA isolation, and cDNA generation*

Sequence information for the genes of interest - *mnsod*, *fesod*, *apx*, and *katg* - was generated from available monoclonal batch cultures of a range of *Symbiodinium* types, and the sequence results cross-checked with existing EST libraries for some of these types (Table 3). Especially in the case of *fesod*, amplifications were not successful for all types. EST libraries also served as a complementary source of additional isoforms, where applicable.

**Table 3:** **Sample and sequence information**

**ITS2 type**	**Culture ID**	**Species**	**Isolated from**	**Sample origin**	**Data source**	**Reference**
A1	CCMP2467	*Symbiodinium microadriaticum subsp. microadriaticum*	*Stylophora pistillata*	Red Sea	EST library	[90]
A1	CCMP2467	*Symbiodinium microadriaticum subsp. microadriaticum*	*Stylophora pistillata*	Red Sea	PCR amplification/conceptual translation	this study
A1	Casskb8	*Symbiodinium microadriaticum*	*Cassiopea xamachana*	Hawai’i	EST library	[45,91]
B1	Mf1.05b	*Symbiodinium minutum*	*Orbicella faveolata*	Florida Keys	EST library	[45]
B1	Ap1	*Symbiodinium minutum*	*Aiptasia pulchella*	Hawai’i	PCR amplification /conceptual translation	this study
C1	CCMP2466	*Symbiodinium goreauii*	*Discosoma sanctithomae*	Jamaica	PCR amplification /conceptual translation	this study
C3	N/A	N/A	*Acropora aspera*	Great Barrier Reef	EST library	[46]
C3	Mp	N/A	*Mastigias papua*	Palau	PCR amplification /conceptual translation	this study
C15	N/A	N/A	*Montipora digitata*	Great Barrier Reef	PCR amplification /conceptual translation	this study
D	N/A	N/A	*Acropora hyacinthus*	American Samoa	EST library	[48]
E	CCMP421	*Symbiodinium voratum*	free-living	New Zealand	PCR amplification /conceptual translation	this study
F1	CCMP2468	*Symbiodinium kawagutii*	*Montipora capitata*	Hawai’i	EST library	[47]
F1	Mv	*Symbiodinium kawagutii*	*Montipora capitata*	Hawai’i	PCR amplification /conceptual translation	this study

*Symbiodinium* types, biogeographical origin and source of antioxidant sequence data analysed in this study. The culture identification number (ID, when applicable), formal species name, source of isolation, geographic origin, and the sequencing method have been included. ITS2 = internal transcribed spacer 2; EST = expressed sequence tag; N/A = not applicable.

Total RNA from 10 mL of a pelleted batch culture (2000 x *g*, 5 min) from the *Symbiodinium* ITS2 types A1, B1, C1, C3, C15, E, and F1 (Table 3) was extracted with a bead mill (50 Hz, 5 min, 4°C), using the Purelink® RNA Mini Kit (Life technologies; includes DNAse treatment). cDNA was generated by reverse transcription (QuantiTect Reverse Transcription Kit, Qiagen; includes DNAse treatment) according to the manufacturer’s instructions. Quantity and quality of extracted RNA was verified via spectrophotometric analysis (Nanodrop 1000, Thermo Fisher Scientific). All *Symbiodinium* identities were based on ITS2 sequencing and were performed as published previously [92].

#### Primer design and amplification strategy

Amino acid sequences for the genes of interest - *mnsod*, *fesod*, *apx*, and *katg* - from closely related taxa were obtained from GenBank [93], Uniprot [94] and Peroxibase [95], and searched against the *Symbiodinium* C3 EST database [46]. Matching contigs were aligned to obtain consensus sequences for the coding region of interest. Multiple primers selected at random locations across the consensus were generated for each sequence using the Primer3 [96] plug-in in Geneious® 6.1.8 (Biomatters Ltd., New Zealand) and tested with cDNA derived from different *Symbiodinium* ITS2 types (Additional file 9). In addition, a spliced leader primer (SL-primer; 5′-CCGTAGCCATTTTGGCTCAAG-3′; [97]) was tested in conjunction with working reverse primers in an attempt to amplify the N-terminal coding region of each gene.

Primers were tested using a general PCR amplification profile, consisting of an initial denaturation of 3 min at 95°C, followed by 35 cycles of 30 s at 95°C, 30 s at 54°C and 1 min at 72°C with a final elongation of 7 min at 72°C, using MyTaq^TM^ Red Mix (BIOLINE). PCR amplicons were visualized via gel electrophoresis (1.5-3.5% [w/v] agarose, using the molecular ladders Hyperladder II or V [BIOLINE] for estimation of amplicon size). Successful amplifications were purified using ExoSAP-IT (Global Science) and sequenced in both directions by Macrogen Inc. (Seoul, South Korea). When non-specific amplifications occurred, annealing temperatures were raised to 60°C to increase PCR specificity and/or bands were manually excised and purified prior to sequencing (Zymoclean^TM^ Gel DNA Recovery Kit, according to manufacturer’s recommendations).

#### Identification, alignment and annotation of coding sequences

Partial sequences for each gene of interest and *Symbiodinium* type were aligned using Clustal W [98], and the consensus sequences were searched against GenBank (Blastx) [99] for verification of identity. In addition, these sequences were searched (Blastn) against available EST libraries for *Symbiodinium* types A1, B1, C3, D, and F1 in order to obtain additional sequences (Table 3).

Sequences were analysed for the presence of open reading frames (ORF) using ATGpr software [100] and translated accordingly. Amino acid sequences were analysed using Phobius [101] for the presence of signal peptides and transmembrane domains, TargetP 1.1 [102] for prediction of subcellular localisation, and GPI-SOM [103] and PredGPI [104] for the presence of a glypiation site (posttranslational attachment of glycophosphatidylinositol [GPI membrane anchor]). 3-D models, based on the mature (without N-terminal signal) full-length amino acid sequence were developed using i-tasser [105]. Alignment of 3-D models and visual highlighting of specific features were performed in Geneious® 6.1.8.

#### Phylogenetic analyses

Sequences were aligned with Clustal W and trimmed to equal lengths. Appropriate phylogenetic models for protein evolution were determined with ProtTest using the Akaike Information Criterion [106]. Tree topology was determined using maximum likelihood (ML) analysis with 100 bootstraps to infer topology robustness [107,108]. Phylogenetic trees were generated using the PhyML [109] plug-in in Geneious® 6.1.8 and node supports >70 highlighted where appropriate.

### Gene expression experiment

#### Experimental setup

For details on *Symbiodinium* cell culture and experimental methodology see Additional file 10. Briefly, batch cultures of *Symbiodinium* type B1 (culture ID Ap1; N = 6 per treatment), grown at 25°C and 40–50 μmol quanta m^−2^ s^−1^ (LI-COR Quantum light meter LI-189 with cosine sensor, LI-COR, Inc., USA) were exposed to 33°C over three days after rapid heating (1°C h^−1^). This *Symbiodinium* type was chosen, because previous experiments have indicated a high degree of thermal susceptibility under the experimental setup employed here [36]. Samples were taken on Days 0, 1 and 3 by pelleting seven 50 mL aliquots (2000 x *g*, 5 min) that were flash frozen in liquid nitrogen and stored at −80°C. In addition, 5–10 mL aliquots were taken for determination of maximum quantum yield of photosystem II (F_v_/F_m_) via PAM fluorometry (Water-PAM chlorophyll fluorometer, Heinz Walz GmbH, Germany) and measurement of cell density via haemocytometer counts (see Additional file 10).

#### Viability and antioxidant activity

Rates of asexual reproduction, F_v_/F_m_ and chlorophyll *a* content per cell were monitored as proxies for overall cell viability (see Additional file 10 for methodological details). The molecular response was monitored via activity of SOD, APX and KatG, and all measurements were conducted according to previously published protocols (see Additional file 10). Enzyme activities were normalized per cell and expressed as specific activity (U cell^−1^), where one unit catalyses one μmol substrate min^−1^ cell^−1^.

#### Housekeeping genes (HKG) and genes of interest (GOI) for *Symbiodinium* B1

After pooling three frozen pellets per replicate and time-point, RNA was extracted and converted to cDNA as described above. The housekeeping genes (HKGs) ß-tubulin (*tub*), S-adenosyl methionine synthetase (*sam*), calmodulin (*cal*), and cytochrome oxidase subunit 1 (*cox*) were chosen based on a previous study [82], with *fesod*, *mnsod*, *apx* and *katg* as genes of interest (GOI). Partial sequences for the HKGs that allowed the design of qPCR primers were obtained by either combining the SL-primer with reverse HKG primers previously used for *Symbiodinium* C3 [82], or amplified based on EST sequences for *Symbiodinium* B1 (Table 3). All qPCR primer pairs were designed with the Primer3 plug-in of Geneious® 6.1.8, with annealing temperatures of 60°C.

#### Quantitative polymerase chain reaction (qPCR)

HKG and GOI qPCR primers (Table 4) were chosen as the result of the following optimization procedure. The efficiency and specificity of different designed HKG and GOI qPCR primers were tested with a pooled cDNA sample (from all six *Symbiodinium* B1 replicates from 25°C/Day 0). Equimolar primer pair concentrations in the range of 200–1000 nM were tested for each pair and efficiency was assessed using a template dilution series (five dilution levels from 1:5–1:500). Specificity and product size were assessed via melt curve analysis and gel electrophoresis.

**Table 4:** **Primer properties**

**Name**	**Gene**	**Forward primer (5′-3′)**	**Reverse primer (5′-3′)**	**Amplicon size [bp]**	**Concentration [nM]**	**Efficiency**
Calmodulin (HKG)	*cal*	TGATGGCGCGCAAGATGAAGG	TGCCATCGCGATCGAAAACCTTG	78	750	98%
Cytochrome oxidase subunit 1 (HKG)	*cox*	TCTGTCTTCCTCTCACATCTCT	CCACTGCACCATTTCCAAGA	82	225	97%
S-adenosyl methionine synthetase (HKG)	*sam*	GACCAAGAACGGCATCAAGT	TGCTGCTCATGGATGCATAC	74	200	95%
ß-tubulin (HKG)	*tub*	CCAGCTTTGCCATTCCCTTG	TGGTTCCACCACTGTGTCAG	148	750	94%
Hybrid ascorbate-cytochrome *c* peroxidase (GOI)	*apx1*	CAATGTGGCACTCATGCTGG	TAAGCTTCTCAAGGTCCGCC	107	500	102%
Catalase peroxidase (GOI)	*katg1*	TCTTCTTGGCCAAGTGAAGC	TTTGATGGCAGTGGTTCCTG	85	500	96%
Manganese superoxide dismutase (GOI)	*mnsod1 + 2*	CAACCCCAAACCAGGACAAT	CACATCCCACCAAGCTTTGA	146	1000	96%

Primer sequences, amplicon size, equimolar primer concentrations and efficiency for housekeeping genes (HKGs) and antioxidant genes of interest (GOI) used for quantitative real-time PCR-based (SYBR® Green) gene expression in *Symbiodinium* type B1. The isoform designations for the antioxidant genes are based on results presented in this study.

Real-time polymerase chain reactions (qPCRs, three per sample) were performed with 2 μL of 1:50 diluted template in 20 μL reaction volume, using a Power SYBR® Green Master Mix (Life Technologies). qPCRs were run on a StepOne^TM^ Real-Time PCR machine (Applied Biosystems, USA) and consisted of an initial incubation for 10 min at 95°C, followed by 40 cycles of 15 s at 95°C and 1 min at 60°C. The run was concluded with a melt curve from 60°C to 95°C. All sample C_t_-values were within the range of the template dilution series used to assess efficiency. Despite testing a number of *fesod* qPCR primer pairs, it was not possible to consistently assess the expression of this gene due to multiple peaks in the melting curve or low efficiencies.

### Statistical analysis

Physiological variables were analysed for temperature and time effects using repeated measures analysis of variance (rmANOVA). Cell densities were log transformed and F_v_/F_m_ values were arcsine square root transformed. Datasets were tested for sphericity with Mauchly’s sphericity test. The results of Pillai’s trace test or epsilon-adjusted univariate F-tests (Greenhouse-Geisser; G-G) at a confidence level of 0.05 are reported. Reported *post hoc* contrasts were adjusted for multiple comparisons, using the Bonferroni correction. Data were analysed using JMP 10.0.0 (SAS Institute Inc., USA).

Stable HKGs were identified by comparing HKG expression at 25°C and 33°C over time using the software Normfinder [110]. Relative gene expression was calculated from C_t_-values, using REST 2009 with 10000 iterations [111].

### Availability of supporting data

Primary references for used *Symbiodinium* EST libraries are given in Table 3. The complete list of used contigs (contig designations are library-specific) and derived assemblies, as well as NCBI accession numbers (where applicable) are given in Additional file 11. A list of used primers to amplify antioxidant cDNA fragments is provided in Additional file 9. All explicit amino acid sequences are provided in Figures 1, 3 and 5 and available in txt format in Additional files 12, 13, and 14.

## Additional files

Additional file 1:
**SOD pairwise amino acid identities.** Pairwise amino acid sequence identities of MnSOD (#1-21) and FeSOD (#22-25) sequences from different *Symbiodinium* ITS2 types based on the alignment in Figure 1. Pairwise identities between full length sequences (green) and pairwise identities within clade C (yellow) have been highlighted. Additional file 2:
**MnSOD signal peptide location.** Location of signal peptide (magenta) and ancient transit peptide motif (red) in the N-terminal region of MnSOD sequences from different *Symbiodinium* ITS2 types. Sequence IDs consist of ITS2 type, strain designation or source of isolation (in brackets), MnSOD isoform and NCBI accession number or contig/assembly designation (Additional file 11). Additional file 3:
**Classification of** ***Symbiodinium*** **peroxidases.** “Peroxiscan” classification and BLAST (blastp) results for different *Symbiodinium* peroxidase isoforms (http://peroxibase.toulouse.inra.fr). Additional file 4:
**APX signal peptide location.** Location of signal peptide (magenta) and transmembrane domains (red) in the N-terminal region of APX sequences from different *Symbiodinium* ITS2 types. Sequence IDs consist of ITS2 type, strain designation or source of isolation (in brackets), APX isoform and NCBI accession number or contig/assembly designation (Additional file 11). Additional file 5:
**APX pairwise amino acid identities.** Pairwise amino acid sequence identities of APX sequences from different *Symbiodinium* ITS2 types based on the alignment in Figure 3. Pairwise identities between full length sequences (green) and pairwise identities within clade C (yellow) have been highlighted. Additional file 6:
**KatG signal peptide location.** Location of signal peptide (magenta) and transmembrane domains (red) in the N-terminal region of KatG sequences from different *Symbiodinium* ITS2 types. Sequence IDs consist of ITS2 type, strain designation or source of isolation (in brackets), APX isoform and NCBI accession number or contig/assembly designation (Additional file 11). Additional file 7:
**KatG pairwise amino acid identities.** Pairwise amino acid sequence identities of KatG sequences from different *Symbiodinium* ITS2 types based on the alignment in Figure 5. Pairwise identities between full length sequences (green) and pairwise identities within clade C (yellow) have been highlighted. Additional file 8:
**Location of SymKatG inserts.** Schematization of KatG protein alignment used for the phylogenetic analysis (Figure 10). Size and location of SymKatG inserts relative to other organisms are indicated in blue. Shading indicates site-specific similarity over all sequences as 100% (black), 80-100% (dark grey), 60-80% (light grey), and less than 60% (white), based on the Blosum62 score matrix with a threshold of 1. *Symbiodinium* sequence IDs consist of ITS2 type, strain designation or source of isolation (in brackets), KatG isoform and NCBI accession number or contig/assembly designation (Additional file 11). Additional file 9:
**Primer list. Primer sequences of successful fragment amplifications, used for assemblies of antioxidant genes of interest.** Sequence IDs contain ITS2 type, strain designation or source of isolation (in brackets) and GenBank accession number. Additional file 10:
**Supplementary Methods.** Detailed information on experimental setup, PAM fluorometry and growth measurements, sample processing and enzyme measurement for gene expression experiment. Additional file 11:
**Sequence details.** List of biogeographic origin and sequence IDs and accession numbers of used contigs for all antioxidant sequences presented in this study. New sequences generated in this study are highlighted (*). Additional file 12:
**SOD alignment.** *Symbiodinium* superoxide dimutase (Mn/Fe) amino acid alignment (Figure 1) in txt format. Additional file 13:
**APX alignment.** *Symbiodinium* ascorbate peroxidase amino acid alignment (Figure 3) in txt format. Additional file 14:
**KatG alignment.** *Symbiodinium* catalase peroxidase amino acid alignment (Figure 5) in txt format.

## Abbreviations

APX: Ascorbate peroxidase
APX-CcP: Hybrid ascorbate-cytochrome *c* peroxidases
Cal: Calmodulin
Chl *a*: Chlorophyll *a*
Cox: Cytochrome oxidase subunit 1
EST: Expressed sequence tags
FeSOD: Iron superoxide dismutase
F_v_/F_m_: Maximum quantum yield of photosystem II
G-G: Greenhouse-Geisser
GOI: Gene of interest
GPI: Glycosylphosphatidylinositol
HKG: House-keeping gene
ITS2: Internal transcribed spacer 2
KatG: Catalase peroxidase
MnSOD: Manganese superoxide dismutase
mRNA: Messenger ribonucleic acid
ORF: Open reading frame
OTU: Operational taxonomic unit
PDB: Protein data bank
qPCR: Real-time polymerase chain reaction
rDNA: Ribosomal deoxyribonucleic acid
rmANOVA: Repeated measures analysis of variance
ROS: Reactive oxygen species
SAM: S-adenosyl methionine synthetase
SAR: Stramenopile-Alveolata-Rhizaria
SOD: Superoxide dismutase
Tub: ß-tubulin
