# Supplementary material for: Transcriptomic characterization of the enzymatic antioxidants FeSOD, MnSOD, APX and KatG in the dinoflagellate genus Symbiodinium
Source: BMC Evol Biol. 2015 Mar 18;15:48. doi: 10.1186/s12862-015-0326-0 (PMC4416395; doi:10.1186/s12862-015-0326-0)
Supplement: Additional file 1: — SOD pairwise amino acid identities. Pairwise amino acid sequence identities of MnSOD (#1-21) and FeSOD (#22-25) sequences from different Symbiodinium ITS2 types based on the alignment in Figure 1. Pairwise identities between full length sequences (green) and pairwise identities within clade C (yellow) have been highlighted. [file 12862_2015_326_MOESM1_ESM.pdf]

		Full length	Ungapped length [aa]	B1 Ap1 SymMnSOD1	B1 Mf1.05b SymMnSOD1 Assembly2	C1 CCMP2466 SymMnSOD1	C3 Mp SymMnSOD1 AHI54358	C3 A.aspera SymMnSOD1 FE866047	C15 M.digitata SymMnSOD1	B1 Mf1.05b SymMnSOD2 Assembly1	B1 Mf1.05b SymMnSOD2 rep_c13368	D A.hyacinthus SymMnSOD2 GAFP01017905	F1 CCMP2468 SymMnSOD2 Assembly1	A1 Casskb8 SymMnSOD3 rep_c4192	A1 CCMP2467 SymMnSOD3 Assembly2	A1 CCMP2467 SymMnSOD3 Assembly6	B1 Mf1.05b SymMnSOD3 _c19510	D A.hyacinthus SymMnSOD3 GAFP01006955	A1 Casskb8 MnSOD rep_c710	A1 CCMP2467 MnSOD Assembly4	A1 CCMP2467 MnSOD Assembly3	A1 Casskb8 MnSOD _c18645	A1 CCMP2467 MnSOD Assembly1	B1 Mf1.05b MnSOD _c29099	A PF-2005 SymFeSOD AY916504	B1 Ap1 SymFeSOD KJ672519	E CCMP421 SymFeSOD KJ672517	F1 Mv SymFeSOD KJ672518
				1	2	3	4	5	6	7	8	9	10	11	12	13	14	15	16	17	18	19	20	21	22	23	24	25
		Full length			Y	Y	Y		Y	Y	Y	Y	Y	Y				Y	Y						Y			
			Ungapped length [aa]	261	265	263	265	105	265	299	303	294	297	246	173	86	86	247	270	189	80	281	193	148	201	148	149	132
B1 Ap1 SymMnSOD1 KJ672521	1		261		99%	92%	94%	92%	93%	96%	96%	85%	93%	49%	35%	60%	60%	44%	79%	76%	87%	83%	81%	41%				
B1 Mf1.05b SymMnSOD1 Assembly2	2	Y	265	99%		91%	93%	93%	93%	95%	95%	84%	92%	48%	35%	57%	57%	44%	79%	76%	86%	82%	80%	41%				
C1 CCMP2466 SymMnSOD1 AHI54358	3	Y	263	92%	91%		96%	97%	96%	94%	94%	86%	94%	49%	35%	60%	60%	45%	80%	76%	89%	84%	80%	40%				
C3 Mp SymMnSOD1 KJ672520	4	Y	265	94%	93%	96%		98%	97%	95%	95%	87%	96%	49%	35%	59%	59%	45%	80%	77%	86%	84%	81%	41%				
C3 A.aspera SymMnSOD1 FE866047	5		105	92%	93%	97%	98%		96%	93%	93%	93%	95%	55%	29%	59%	59%	52%	89%	96%	86%	89%	87%	41%				
C15 M.digitata SymMnSOD1 KJ672522	6	Y	265	93%	93%	96%	97%	96%		95%	95%	90%	96%	49%	36%	59%	59%	45%	81%	77%	88%	85%	82%	41%				
B1 Mf1.05b SymMnSOD2 Assembly1	7	Y	299	96%	95%	94%	95%	93%	95%		97%	88%	95%	47%	35%	52%	52%	43%	81%	78%	88%	85%	82%	40%				
B1 Mf1.05b SymMnSOD2 rep_c13368	8	Y	303	96%	95%	94%	95%	93%	95%	97%		87%	93%	47%	35%	52%	52%	43%	81%	77%	88%	85%	82%	40%				
D A.hyacinthus SymMnSOD2 GAFP01017905	9	Y	294	85%	84%	86%	87%	93%	90%	88%	87%		89%	47%	35%	52%	52%	43%	82%	78%	89%	84%	81%	41%				
F1 CCMP2468 SymMnSOD2 Assembly1	10	Y	297	93%	92%	94%	96%	95%	96%	95%	93%	89%		47%	35%	53%	53%	43%	80%	77%	88%	85%	83%	40%				
A1 Casskb8 SymMnSOD3 rep_c4192	11	Y	246	49%	48%	49%	49%	55%	49%	47%	47%	47%	47%		76%	100%	100%	73%	49%	43%	61%	48%	46%	66%				
A1 CCMP2467 SymMnSOD3 Assembly2	12		173	35%	35%	35%	35%	29%	36%	35%	35%	35%	35%	76%		43%	43%	53%	36%	39%	28%	37%	42%	61%				
A1 CCMP2467 SymMnSOD3 Assembly6	13		86	60%	57%	60%	59%	59%	59%	52%	52%	52%	53%	100%	43%		100%	92%	63%	N/A	63%	51%	N/A	N/A				
B1 Mf1.05b SymMnSOD3 _c19510	14		86	60%	57%	60%	59%	59%	59%	52%	52%	52%	53%	100%	43%	100%		92%	63%	N/A	63%	51%	N/A	N/A				
D A.hyacinthus SymMnSOD3 GAFP01006955	15	Y	247	44%	44%	45%	45%	52%	45%	43%	43%	43%	43%	73%	53%	92%	92%		47%	42%	58%	44%	43%	70%				
A1 Casskb8 MnSOD rep_c710	16	Y	270	79%	79%	80%	80%	89%	81%	81%	81%	82%	80%	49%	36%	63%	63%	47%		100%	100%	82%	80%	41%				
A1 CCMP2467 MnSOD Assembly4	17		189	76%	76%	76%	77%	96%	77%	78%	77%	78%	77%	43%	39%	N/A	N/A	42%	100%		N/A	80%	80%	41%				
A1 CCMP2467 MnSOD Assembly3	18		80	87%	86%	89%	86%	86%	88%	88%	88%	89%	88%	61%	28%	63%	63%	58%	100%	N/A		86%	80%	N/A				
A1 Casskb8 MnSOD _c18645	19		281	83%	82%	84%	84%	89%	85%	85%	85%	84%	85%	48%	37%	51%	51%	44%	82%	80%	86%		100%	43%				
A1 CCMP2467 MnSOD Assembly1	20		193	81%	80%	80%	81%	87%	82%	82%	82%	81%	83%	46%	42%	N/A	N/A	43%	80%	80%	80%	100%		43%				
B1 Mf1.05b MnSOD _c29099	21		148	41%	41%	40%	41%	41%	41%	40%	40%	41%	40%	66%	61%	N/A	N/A	70%	41%	41%	N/A	43%	43%					
A PF-2005 SymFeSOD AY916504	22	Y	201																							95%	94%	95%
B1 Ap1 SymFeSOD KJ672519	23		148																						95%		96%	96%
E CCMP421 SymFeSOD KJ672517	24		149																						94%	96%		100%
F1 Mv SymFeSOD KJ672518	25		132																						95%	96%	100%	
