# Supplementary material for: Transcriptomic characterization of the enzymatic antioxidants FeSOD, MnSOD, APX and KatG in the dinoflagellate genus Symbiodinium
Source: BMC Evol Biol. 2015 Mar 18;15:48. doi: 10.1186/s12862-015-0326-0 (PMC4416395; doi:10.1186/s12862-015-0326-0)
Supplement: Additional file 2: — MnSOD signal peptide location. Location of signal peptide (magenta) and ancient transit peptide motif (red) in the N-terminal region of MnSOD sequences from different Symbiodinium ITS2 types. Sequence IDs consist of ITS2 type, strain designation or source of isolation (in brackets), MnSOD isoform and NCBI accession number or contig/assembly designation (Additional file 11). [file 12862_2015_326_MOESM2_ESM.pdf]

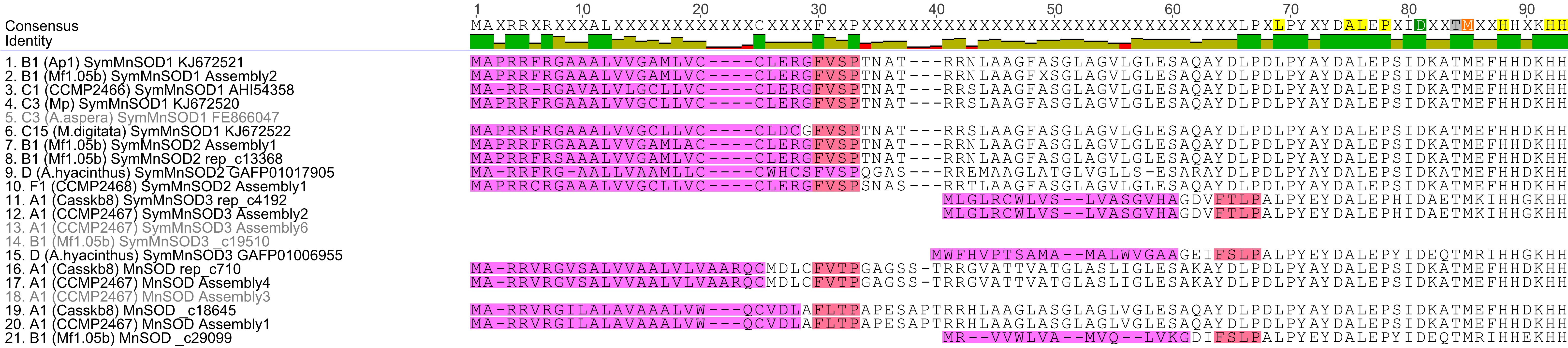

1 10 20 30 40 50 60 70 80 90
Consensus MAXRRXRXXXALXXXXXXXXXXXXCXXXXFXXPXXXXXXXXXXXXXXXXXXXXXXXXXXXXXXXXLPXLPYXYDALEPXIDXXTMXXHHXKHH
Identity
1. B1 (Ap1) SymMnSOD1 KJ672521 MAPRRFRGAAALVVGAMLVC----CLERGFVSPTNAT---RRNLAAGFASGLAGVLGLESAQAYDLPDLPYAYDALEPSIDKATMEFHHDKHH
2. B1 (Mf1.05b) SymMnSOD1 Assembly2 MAPRRFRGAAALVVGAMLVC----CLERGFVSPTNAT---RRNLAAGFXSGLAGVLGLESAQAYDLPDLPYAYDALEPSIDKATMEFHHDKHH
3. C1 (CCMP2466) SymMnSOD1 AHI54358 MA-RR-RGAVALVLGCLLVC----CLERGFVSPTNAT---RRSLAAGFASGLAGVLGLESAQAYDLPDLPYAYDALEPSIDKATMEFHHDKHH
4. C3 (Mp) SymMnSOD1 KJ672520 MAPRRFRGAAALVVGCLLVC----CLERGFVSPTNAT---RRSLAAGFASGLAGVLGLESAQAYDLPDLPYAYDALEPSIDKATMEFHHDKHH
5. C3 (A.aspera) SymMnSOD1 FE866047
6. C15 (M.digitata) SymMnSOD1 KJ672522 MAPRRFRGAAALVVGCLLVC----CLDCGFVSPTNAT---RRSLAAGFASGLAGVLGLESAQAYDLPDLPYAYDALEPSIDKATMEFHHDKHH
7. B1 (Mf1.05b) SymMnSOD2 Assembly1 MAPRRFRGAAALVVGAMLAC----CLERGFVSPTNAT---RRNLAAGFASGLAGVLGLESAQAYDLPDLPYAYDALEPSIDKATMEFHHDKHH
8. B1 (Mf1.05b) SymMnSOD2 rep_c13368 MAPRRFRSAAALVVGAMLVC----CLERGFVSPTNAT---RRNLAAGFASGLAGVLGLESAQAYDLPDLPYAYDALEPSIDKATMEFHHDKHH
9. D (A.hyacinthus) SymMnSOD2 GAFP01017905 MA-RRFRG-AALLVAAMLLC----CWHCSFVSPQGAS---RREMAAGLATGLVGLLS-ESARAYDLPDLPYAYDALEPSIDKATMEFHHDKHH
10. F1 (CCMP2468) SymMnSOD2 Assembly1 MAPRRCRGAAALVVGCLLVC----CLERGFVSPSNAS---RRTLAAGFASGLAGVLGLESAQAYDLPDLPYAYDALEPSIDKATMEFHHDKHH
11. A1 (Casskb8) SymMnSOD3 rep_c4192 MLGLRCWLVS--LVASGVHAGDVFTLPALPYEYDALEPHIDAETMKIHHGKHH
12. A1 (CCMP2467) SymMnSOD3 Assembly2 MLGLRCWLVS--LVASGVHAGDVFTLPALPYEYDALEPHIDAETMKIHHGKHH
13. A1 (CCMP2467) SymMnSOD3 Assembly6
14. B1 (Mf1.05b) SymMnSOD3 _c19510
15. D (A.hyacinthus) SymMnSOD3 GAFP01006955 MWFHVPTSAMA--MALWVGAAGEIFSLPALPYEYDALEPYIDEQTMRIHHGKHH
16. A1 (Casskb8) MnSOD rep_c710 MA-RRVRGVSALVVAALVLVAARQCMDLCFVTPGAGSS-TRRGVATTVATGLASLIGLESAKAYDLPDLPYAYDALEPSIDKATMEFHHDKHH
17. A1 (CCMP2467) MnSOD Assembly4 MA-RRVRGVSALVVAALVLVAARQCMDLCFVTPGAGSS-TRRGVATTVATGLASLIGLESAKAYDLPDLPYAYDALEPSIDKATMEFHHDKHH
18. A1 (CCMP2467) MnSOD Assembly3
19. A1 (Casskb8) MnSOD _c18645 MA-RRVRGILALAVAAALVW---QCVDLAFLTPAPESAPTRRHLAAGLASGLAGLVGLESAQAYDLPDLPYAYDALEPSIDKATMEFHHDKHH
20. A1 (CCMP2467) MnSOD Assembly1 MA-RRVRGILALAVAAALVW---QCVDLAFLTPAPESAPTRRHLAAGLASGLAGLVGLESAQAYDLPDLPYAYDALEPSIDKATMEFHHDKHH
21. B1 (Mf1.05b) MnSOD _c29099 MR--VVWLVA--MVQ--LVKGDIFSLPALPYEYDALEPYIDEQTMRIHHEKHH
