# Supplementary material for: Transcriptomic characterization of the enzymatic antioxidants FeSOD, MnSOD, APX and KatG in the dinoflagellate genus Symbiodinium
Source: BMC Evol Biol. 2015 Mar 18;15:48. doi: 10.1186/s12862-015-0326-0 (PMC4416395; doi:10.1186/s12862-015-0326-0)
Supplement: Additional file 3: — Classification of Symbiodinium peroxidases. “Peroxiscan” classification and BLAST (blastp) results for different Symbiodinium peroxidase isoforms (http://peroxibase.toulouse.inra.fr). [file 12862_2015_326_MOESM3_ESM.pdf]

Table S1. “Peroxiscan” classification and BLAST (blastp) results for different *Symbiodinium* peroxidase isoforms (http://peroxibase.toulouse.inra.fr).

Isoform	Peroxibase class	Top BLAST hit	Peroxibase entry ID	Identity	E-value
SymAPX1	Hybrid ascorbate-cytochrome *c* peroxidase	*Thalassiosira pseudonana*	2557	56-61%	10^{-101} to 10^{-107}
SymAPX2	Hybrid ascorbate-cytochrome *c* peroxidase	*Thalassiosira pseudonana*	2557	73-74%	10^{-136} to 10^{-142}
SymAPX3	Ascorbate peroxidase	*Karlodinium micrum*	3944	72-73%	10^{-164} to 10^{-167}
SymAPX4	Ascorbate peroxidase	*Karlodinium micrum*	3944	62%	10^{-128}
SymAPX5	Ascorbate peroxidase	*Karlodinium micrum*	3944	56%	10^{-116} to $1E^{-118}$
SymAPX6	Ascorbate peroxidase related	*Thalassiosira pseudonana*	2559	37%	10^{-47}
SymKatG1	Catalase peroxidase	*Volvox carteri*	8373	36%	10^{-80}
SymKatG2	Catalase peroxidase	*Karenia brevis*	2527	53%	10^{-153}
