# Supplementary material for: Transcriptomic characterization of the enzymatic antioxidants FeSOD, MnSOD, APX and KatG in the dinoflagellate genus Symbiodinium
Source: BMC Evol Biol. 2015 Mar 18;15:48. doi: 10.1186/s12862-015-0326-0 (PMC4416395; doi:10.1186/s12862-015-0326-0)
Supplement: Additional file 4: — APX signal peptide location. Location of signal peptide (magenta) and transmembrane domains (red) in the N-terminal region of APX sequences from different Symbiodinium ITS2 types. Sequence IDs consist of ITS2 type, strain designation or source of isolation (in brackets), APX isoform and NCBI accession number or contig/assembly designation (Additional file 11). [file 12862_2015_326_MOESM4_ESM.pdf]

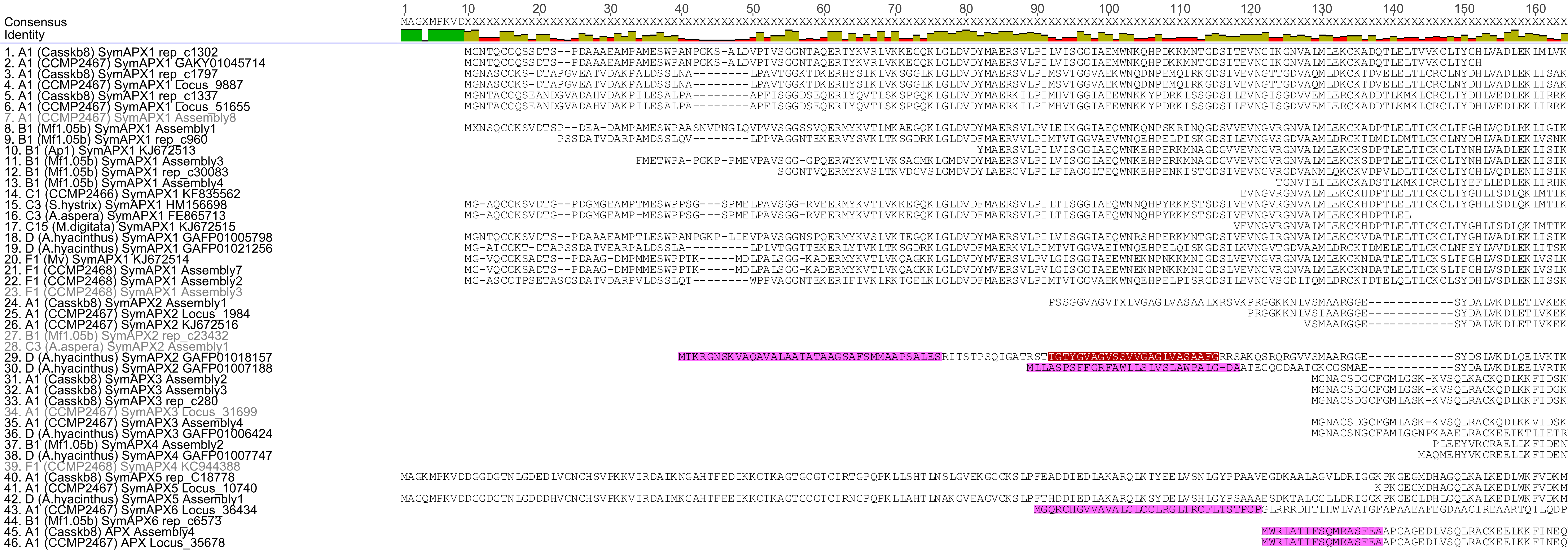
1 10 20 30 40 50 60 70 80 90 100 110 120 130 140 150 160
Consensus MAGXMPKVDXXXXXXXXXXXXXXXXXXXXXXXXXXXXXXXXXXXXXXXXXXXXXXXXXXXXXXXXXXXXXXXXXXXXXXXXXXXXXXXXXXXXXXXXXXXXXXXXXXXXXXXXXXXXXXXXXXXXXXXXXXXXXXXXXXXXXXXXXXXXXXXXXXX
Identity
1. A1 (Casskb8) SymAPX1 rep_c1302 MGNTQCCQSSDTS--PDAAAEAMPAMESWPANPGKS-ALDVPTVSGGNTAQERTYKVRLVKKEGQKLGLDVDYMAERSVLPILVISGGIAEMWNKQHPDKKMNTGDSITEVNGIKGNVALMLEKCKADQTLELTVVKCLTYGHLVADLEKLMLVK
2. A1 (CCMP2467) SymAPX1 GAKY01045714 MGNTQCCQSSDTS--PDAAAEAMPAMESWPANPGKS-ALDVPTVSGGNTAQERTYKVRLVKKEGQKLGLDVDYMAERSVLPILVISGGIAEMWNKQHPDKKMNTGDSITEVNGIKGNVALMLEKCKADQTLELTVVKCLTYGH
3. A1 (Casskb8) SymAPX1 rep_c1797 MGNASCCKS-DTAPGVEATVDAKPALDSSLNA--------LPAVTGGKTDKERHYSIKLVKSGGLKLGLDVDYMAERSVLPIMSVTGGVAEKWNQDNPEMQIRKGDSIVEVNGTTGDVAQMLDKCKTDVELELTLCRCLNYDHLVADLEKLISAK
4. A1 (CCMP2467) SymAPX1 Locus_9887 MGNASCCKS-DTAPGVEATVDAKPALDSSLNA--------LPAVTGGKTDKERHYSIKLVKSGGLKLGLDVDYMAERSVLPIMSVTGGVAEKWNQDNPEMQIRKGDSIVEVNGTTGDVAQMLDKCKTDVELELTLCRCLNYDHLVADLEKLISAK
5. A1 (Casskb8) SymAPX1 rep_c1337 MGNTACCQSEANDGVADAHVDAKPILESALPA--------APFISGGDSEQERIYQVTLSKSPGQKLGLDVDYMAERKILPIMHVTGGIAEEWNKKYPDRKLSSGDSILEVNGISGDVVEMLERCKADDTLKMKLCRCLTYDHLVEDLEKLIRRK
6. A1 (CCMP2467) SymAPX1 Locus_51655 MGNTACCQSEANDGVADAHVDAKPILESALPA--------APFISGGDSEQERIYQVTLSKSPGQKLGLDVDYMAERKILPIMHVTGGIAEEWNKKYPDRKLSSGDSILEVNGISGDVVEMLERCKADDTLKMKLCRCLTYDHLVEDLEKLIRRK
7. A1 (CCMP2467) SymAPX1 Assembly8
8. B1 (Mf1.05b) SymAPX1 Assembly1 MXNSQCCKSVDTSP--DEA-DAMPAMESWPAASNVPNGLQVPVVSGGSSVQERMYKVTLMKAEGQKLGLDVDYMAERSVLPVLEIKGGIAEQWNKQNPSKRINQGDSVVEVNGVRGNVALMLEKCKADPTLELTICKCLTFGHLVQDLRKLIGIK
9. B1 (Mf1.05b) SymAPX1 rep_c960 PSSDATVDARPAMDSSLQV--------LPPVAGGNTEKERVYSVKLTKSGDRKLGLDVDFMAERVVLPIMTVTGGVAEVWNQEHPELPISKGDSILEVNGVSGDVAAMLDRCKTDMDLDMTLCKCLNYDHLVADLEKLVSNK
10. B1 (Ap1) SymAPX1 KJ672513 YMAERSVLPILVISGGLAEQWNKEHPERKMNAGDGVVEVNGVRGNVALMLEKCKSDPTLELTICKCLTYNHLVADLEKLISIK
11. B1 (Mf1.05b) SymAPX1 Assembly3 FMETWPA-PGKP-PMEVPAVSGG-GPQERWYKVTLVKSAGMKLGMDVDYMAERSVLPILVISGGLAEQWNKEHPERKMNAGDGVVEVNGVRGNVALMLEKCKSDPTLELTICKCLTYNHLVADLEKLISIK
12. B1 (Mf1.05b) SymAPX1 rep_c30083 SGGNTVQERMYKVSLTKVDGVSLGMDVDYLAERCVLPILFIAGGLTEQWNKEHPENKISTGDSIVEVNGVRGDVANMLQKCKVDPVLDLTICKCLTYGHLVQDLENLISIK
13. B1 (Mf1.05b) SymAPX1 Assembly4 TGNVTEILEKCKADSTLKMKICRCLTYEFLLEDLEKLIRHK
14. C1 (CCMP2466) SymAPX1 KF835562 EVNGVRGNVALMLEKCKHDPTLELTICKCLTYGHLISDLQKLMTIK
15. C3 (S.hystrix) SymAPX1 HM156698 MG-AQCCKSVDTG--PDGMGEAMPTMESWPPSG---SPMELPAVSGG-RVEERMYKVTLVKKEGQKLGLDVDFMAERSVLPILTISGGIAEQWNNQHPYRKMSTSDSIVEVNGVRGNVALMLEKCKHDPTLELTICKCLTYGHLISDLQKLMTIK
16. C3 (A.aspera) SymAPX1 FE865713 MG-AQCCKSVDTG--PDGMGEAMP-MESWPPSG---SPMELPAVSGG-RVEERMYKVTLVKKEGQKLGLDVDFMAERSVLPILTISGGIAEQWNNQHPYRKMSTSDSIVEVNGVRGNVALMLEKCKHDPTLEL
17. C15 (M.digitata) SymAPX1 KJ672515 VEVNGVRGNVALMLEKCKHDPTLELTICKCLTYGHLISDLQKLMTTK
18. D (A.hyacinthus) SymAPX1 GAFP01005798 MGNTQCCKSVDTS--PDAAAEAMPTLESWPANPGKP-LIEVPAVSGGNSPQERMYKVSLVKTEGQKLGLDVDYMAERSVLPILVISGGIAEQWNRSHPERKMNTGDSIVEVNGIRGNVALMLEKCKVDATLELTICKCLTYGHLVADLEKLISIK
19. D (A.hyacinthus) SymAPX1 GAFP01021256 MG-ATCCKT-DTAPSSDATVEARPALDSSLA---------LPLVTGGTTEKERLYTVKLTKSGDRKLGLDVDFMAERKVLPIMTVTGGVAEIWNQEHPELQISKGDSILKVNGVTGDVAAMLDRCKTDMELELTLCKCLNFEYLVVDLEKLITSK
20. F1 (Mv) SymAPX1 KJ672514 MG-VQCCKSADTS--PDAAG-DMPMMESWPPTK-----MDLPALSGG-KADERMYKVTLVKQAGKKLGLDVDYMVERSVLPVLGISGGTAEEWNEKNPNKKMNIGDSLVEVNGVRGNVALMLEKCKNDATLELTLCKSLTFGHLVSDLEKLVSIK
21. F1 (CCMP2468) SymAPX1 Assembly7 MG-VQCCKSADTS--PDAAG-DMPMMESWPPTK-----MDLPALSGG-KADERMYKVTLVKQAGKKLGLDVDYMVERSVLPVLGISGGTAEEWNEKNPNKKMNIGDSLVEVNGVRGNVALMLEKCKNDATLELTLCKSLTFGHLVSDLEKLVSLK
22. F1 (CCMP2468) SymAPX1 Assembly2 MG-ASCCTPSETASGSDATVDARPVLDSSLQT--------WPPVAGGNTEKERIFIVKLRKTGELKLGLDVDFMAERSVLPIMTVTGGVAEKWNQEHPELPISRGDSILEVNGVSGDLTQMLDRCKTDTELQLTLCKCLSYDHLVSDLEKLISSK
23. F1 (CCMP2468) SymAPX1 Assembly3
24. A1 (Casskb8) SymAPX2 Assembly1 PSSGGVAGVTXLVGAGLVASAALXRSVKPRGGKKNLVSMAARGGE------------SYDALVKDLETLVKEK
25. A1 (CCMP2467) SymAPX2 Locus_1984 PRGGKKNLVSIAARGGE------------SYDALVKDLETLVKEK
26. A1 (CCMP2467) SymAPX2 KJ672516 VSMAARGGE------------SYDALVKDLETLVKEK
27. B1 (Mf1.05b) SymAPX2 rep_c23432
28. C3 (A.aspera) SymAPX2 Assembly1
29. D (A.hyacinthus) SymAPX2 GAFP01018157 MTKRGNSKVAQAVALAATATAAGSAFSMMAAPSALESRITSTPSQIGATRSTTGTYGVAGVSSVVGAGLVASAAFGRRSAKQSRQRGVVSMAARGGE------------SYDSLVKDLQELVKTK
30. D (A.hyacinthus) SymAPX2 GAFP01007188 MLLASPSFFGRFAWLLSLVSLAWPALG-DAATEGQCDAATGKCGSMAE------------SYDALVKDLEELVRTK
31. A1 (Casskb8) SymAPX3 Assembly2 MGNACSDGCFGMLGSK-KVSQLKACKQDLKKFIDSK
32. A1 (Casskb8) SymAPX3 Assembly3 MGNACSDGCFGMLGSK-KVSQLRACKQDLKKFIDGK
33. A1 (Casskb8) SymAPX3 rep_c280 MGNACSDGCFGMLASK-KVSQLRACKQDLKKFIDSK
34. A1 (CCMP2467) SymAPX3 Locus_31699
35. A1 (CCMP2467) SymAPX3 Assembly4 MGNACSDGCFGMLASK-KVSQLRACKQDLKKVIDSK
36. D (A.hyacinthus) SymAPX3 GAFP01006424 MGNACSNGCFAMLGGNPKAAELRACKEEIKTLIETR
37. B1 (Mf1.05b) SymAPX4 Assembly2 PLEEYVRCRAELIKFIDEN
38. D (A.hyacinthus) SymAPX4 GAFP01007747 MAQMEHYVKCREELIKFIDEN
39. F1 (CCMP2468) SymAPX4 KC944388
40. A1 (Casskb8) SymAPX5 rep_C18778 MAGKMPKVDDGGDGTNLGDEDLVCNCHSVPKKVIRDAIKNGAHTFEDIKKCTKAGTGCGTCIRTGPQPKLLSHTLNSLGVEKGCCKSLPFEADDIEDLAKARQIKTYEELVSNLGYPPAAVEGDKAALAGVLDRIGGKPKGEGMDHAGQLKAIKEDLWKFVDKM
41. A1 (CCMP2467) SymAPX5 Locus_10740 KPKGEGMDHAGQLKAIKEDLWKFVDKM
42. D (A.hyacinthus) SymAPX5 Assembly1 MAGQMPKVDDGGDGTNLGDDDHVCNCHSVPKKVIRDAIMKGAHTFEEIKKCTKAGTGCGTCIRNGPQPKLLAHTLNAKGVEAGVCKSLPFTHDDIEDLAKARQIKSYDELVSHLGYPSAAAESDKTALGGLLDRIGGKPKGEGLDHLGQLKAIKEDLWKFVDKM
43. A1 (CCMP2467) SymAPX6 Locus_36434 MGQRCHGVVAVALCLCCLRGLTRCFLTSTPCPGLRRRDHTLHWLVATGFAPAAEAFEGDAACIREAARTQTLQDP
44. B1 (Mf1.05b) SymAPX6 rep_c6573
45. A1 (Casskb8) APX Assembly4 MWRLATIFSQMRASFEAAPCAGEDLVSQLRACKEELKKFINEQ
46. A1 (CCMP2467) APX Locus_35678 MWRLATIFSQMRASFEAAPCAGEDLVSQLRACKEELKKFINEQ
