# Supplementary material for: Transcriptomic characterization of the enzymatic antioxidants FeSOD, MnSOD, APX and KatG in the dinoflagellate genus Symbiodinium
Source: BMC Evol Biol. 2015 Mar 18;15:48. doi: 10.1186/s12862-015-0326-0 (PMC4416395; doi:10.1186/s12862-015-0326-0)
Supplement: Additional file 6: — KatG signal peptide location. Location of signal peptide (magenta) and transmembrane domains (red) in the N-terminal region of KatG sequences from different Symbiodinium ITS2 types. Sequence IDs consist of ITS2 type, strain designation or source of isolation (in brackets), APX isoform and NCBI accession number or contig/assembly designation (Additional file 11). [file 12862_2015_326_MOESM6_ESM.pdf]

```
                                            1       10        20        30        40        50        60        70        80        90
Consensus                                   MAXXXXXXXXXXXXXXXXXXXXXXXXXXXXXXXXXXXXXXXXXXXXXXXXXXXXXYXXXLKXLDJXXXXXXXXXLXXXSXXXWPADXXXXX
Identity
1. A1 (Casskb8) SymKatG1 Assembly2
2. A1 (CCMP2467) SymKatG1 KJ735681
3. A1 (CCMP2467) SymKatG1 Assembly3
4. A1 (CCMP2467) SymKatG1 Assembly1                                                                  QVMSKAEYHRQLKSLDIPRLYREIAALMTSSRPYWPADGPQDG
5. B1 (Ap1) SymKatG1 KJ672511                 MALRAAAFLLAFRATDCLMRNDDFPFYDLGTVPAWRDLGSLRGTQSPEQFHKEEYHKQLKKLDIQQLYESITNLMTDSKPYWPADGPQDG
6. B1 (Mf1.05b) SymKatG1 Assembly1            MALRAAAFLLAFRATDCLMRNDDFPFYDLGTVPAWRDLGSLRGTQSPEQFHKEEYHKQLKKLDIQQLYESITNLMTDSKPYWPADGPQDG
7. C1 (CCMP2466) SymKatG1 KF835563           MALRSMGFLLTFLVTEGSMRNDDFPFYYLGTVPAWRDLGSLRGTQSPHQFDKGKYHKQLKELDIEHLYQSISKLMKDSKAFWPADGPQDG
8. C3 (A.aspera) SymKatG1 Assembly1           MAFRSVGFLLTFLVTEGSMRNDDFPFYYLGTVPAWRDLGSLRGTQSPHQFDKEKYHKQLKELDIEHLYQSISKLMKDSKAFWPADGPQDG
9. D (A.hyacinthus) SymKatG1 GAFP1022639
10. D (A.hyacinthus) SymKatG1 GAFP01010732    MAPMFLASLAATVVLSVTAQMRNDDFPFYDLGTIPAWKDLGYLRGTKHAKPFDKEAYHRDLKHLDIPRLYESITKLMVSSKAFWPADGPQDG
11. E (CCMP421) SymKatG1 KJ672510                                                                                                     ADGPQDG
12. F1 (Mv) SymKatG1 KJ672509
13. A1 (Casskb8) SymKatG2 _c15386                              MARKLLAFLVPGVGVWATCPGLPFTTVPENYAAVPELPVYDAALKALDLKAVVTDLQKLFVNSQDCWPADF----
14. A1 (CCMP2467) SymKatG2 Assembly2                            MARKLLAFLVPGVGVWATCPGLPFTTVPENYAAVPELPVYDAALKALDLKAVVTDLQKLFVNSQDCWPADF----
15. B1 (Mf1.05b) SymKatG2 c_32035
16. D (A.hyacinthus) SymKatG2 GAFP01010883                     MARALLLALMLRGSLASPCPGIAYSVPPQNYYVVPELPVYDKSLKELDLKAVVADMQKLFLDSQECWPADW----
```
