# Supplementary figures and images for: Transcriptomic characterization of the enzymatic antioxidants FeSOD, MnSOD, APX and KatG in the dinoflagellate genus Symbiodinium
Source: BMC Evol Biol. 2015 Mar 18;15:48. doi: 10.1186/s12862-015-0326-0 (PMC4416395; doi:10.1186/s12862-015-0326-0)

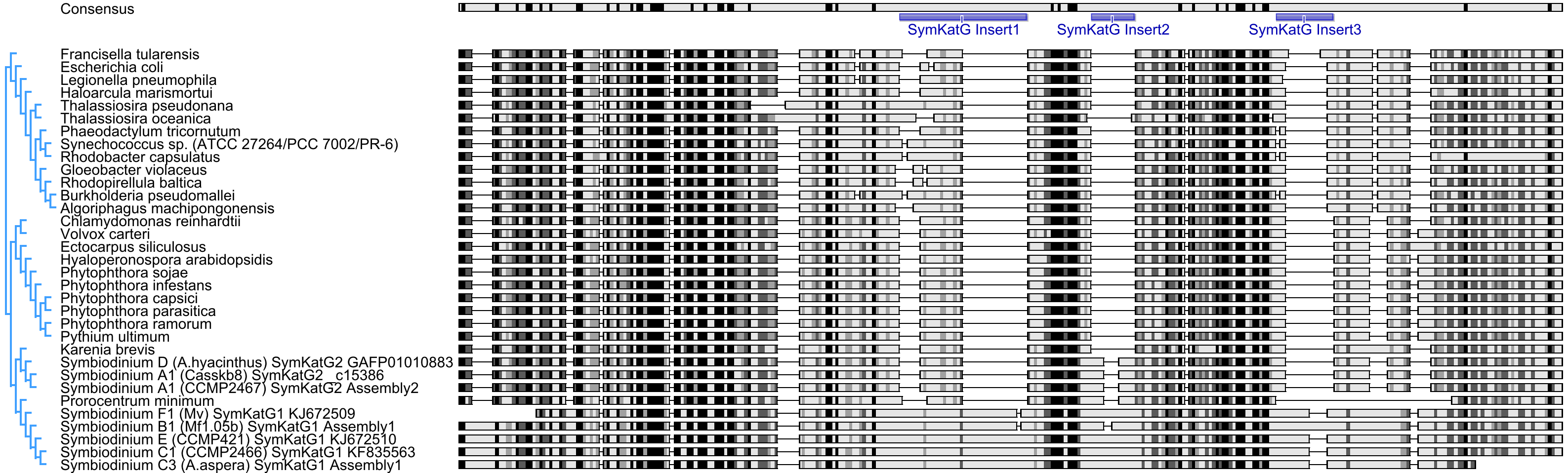

Supplement: Additional file 8: — Location of SymKatG inserts. Schematization of KatG protein alignment used for the phylogenetic analysis (Figure 10). Size and location of SymKatG inserts relative to other organisms are indicated in blue. Shading indicates site-specific similarity over all sequences as 100% (black), 80-100% (dark grey), 60-80% (light grey), and less than 60% (white), based on the Blosum62 score matrix with a threshold of 1. Symbiodinium sequence IDs consist of ITS2 type, strain designation or source of isolation (in brackets), KatG isoform and NCBI accession number or contig/assembly designation (Additional file 11). [file 12862_2015_326_MOESM8_ESM.pdf]
