# Supplementary material for: Transcriptomic characterization of the enzymatic antioxidants FeSOD, MnSOD, APX and KatG in the dinoflagellate genus Symbiodinium
Source: BMC Evol Biol. 2015 Mar 18;15:48. doi: 10.1186/s12862-015-0326-0 (PMC4416395; doi:10.1186/s12862-015-0326-0)
Supplement: Additional file 9: — Primer list. Primer sequences of successful fragment amplifications, used for assemblies of antioxidant genes of interest. Sequence IDs contain ITS2 type, strain designation or source of isolation (in brackets) and GenBank accession number. [file 12862_2015_326_MOESM9_ESM.pdf]

Table S3. Primer sequences of successful fragment amplifications, used for assemblies of antioxidant genes of interest. Sequence IDs contain ITS2 type, strain designation or host (in brackets) and GenBank accession number.

Sequence ID	Forward primer	Sequence (5'-3')	Reverse primer	Sequence (5'-3')
B1 Ap1 SymMnSOD1 KJ672521	SL primer	CCGTAGCCATTTTGGCTCAAG	G4896-077	CACATCCCACCAAGCTTTGA
	G2893-062	CTGGAGCGCGCTTTGGCTCT	G2893-063	ATGCTGCGGGTGCTTTGCCG
C15 (M.digitata) SymMnSOD1 KJ672522	G2946-024	GGTGACAAGAAGCTGGCAAT	G2946-022	ACTGGCTTCAAAGCCCATTC
	G2893-062	CTGGAGCGCGCTTTGGCTCT	G2893-063	ATGCTGCGGGTGCTTTGCCG
	SL primer	CCGTAGCCATTTTGGCTCAAG	G4896-077	CACATCCCACCAAGCTTTGA
	SL primer	CCGTAGCCATTTTGGCTCAAG	G2893-064	GATGTGTGGGAGCATGCCTACTAT
C3 (Mp) SymMnSOD1 KJ672520	SL primer	CCGTAGCCATTTTGGCTCAAG	G2893-064	GATGTGTGGGAGCATGCCTACTAT
	SL primer	CCGTAGCCATTTTGGCTCAAG	G4896-077	CACATCCCACCAAGCTTTGA
B1 Ap1 SymFeSOD KJ672519	G4896-091	CTTCCCGAACTCCCATAT	G2946-028	GATGTGTGGGAGCATGCC
E CCMP421 SymFeSOD KJ672517	G3269-035	GAAACGCTGAACTTCCACCA	G3269-036	TTGGCGAAGTCCCAATTGAC
F1 Mv SymFeSOD KJ672518	G3269-035	GAAACGCTGAACTTCCACCA	G3269-036	TTGGCGAAGTCCCAATTGAC
A1 CCMP2467 SymKatG1 KJ735681	G3269-026	CCTGATCTACGTCTACCCCG	G3269-025	GGTGAGGGAAGCTCTGAGAA
B1 Ap1 SymKatG1 KJ672511	SL primer	CCGTAGCCATTTTGGCTCAAG	G4918-018	TTTGATGGCAGTGGTTCCTG
	SL primer	CCGTAGCCATTTTGGCTCAAG	G3269-028	TGTCCAACCCATCCGATTCT
	G3269-027	TCACCCGAGCAGTTCCATAA	G2893-073	GCCCAGGGCCCTTCAAAGCC
	G3269-027	TCACCCGAGCAGTTCCATAA	G3269-030	GTAGCCCATTTTGTGCCAGT
	G3269-027	TCACCCGAGCAGTTCCATAA	G3269-028	TGTCCAACCCATCCGATTCT
E CCMP421 SymKatG1 KJ672510	G2946-018	AGACTCCAAGGCATTTTGGC	G2946-016	GGATTTGAGCTTTGGCCCTT
	G2893-066	AGGTTGGCATGGCATTGCTCTGG	G2893-067	GCTTTGATTGGCGGTGGGCAC
	G2001-034	TTAGCATCATGGCATTCCGC	G2001-036	ACCGCCAATCAAAGCAACAG
	G2001-039	AACTGTTGCTTTGATTGGCG	G2001-040	AGATAGGTCTGCTGTCAGGC
F1 Mv SymKatG1 KJ672509	G2893-071	AAGCCAGTGGAGGGCCAGCA	G2893-075	GGTGTGCCCACCGCCAATCA
	G2946-018	AGACTCCAAGGCATTTTGGC	G2893-072	TGCTGTCAGGCGCATGGTCG
A1 CCMP2467 SymAPX2 KJ672516	G3269-031	GAGATGAGCATGATGGTGCC	G3269-032	GCAAGAAGAACCTGGTGTCC
B1 Ap1 SymAPX1 KJ672513	G3269-033	TTGGGCATGGATGTGGACTA	G3269-034	TCCTTGTAGTCGGCGAAGAA

C15 M.digitata SymAPX1 KJ672515	G2944-089	CTAGCGACAGCATTGTGGAG	G2944-092	ACATGTCAGGCAGTCAAAGC
	G2944-089	CTAGCGACAGCATTGTGGAG	G2944-090	ACATGCTCCTTGAACTTGGC
	G2893-056	GGAGCGAACGCGGGCTTG	G2893-055	GCACCGGACAGGGCCACAAT
F1 Mv SymAPX1 KJ672514	SL primer	CCGTAGCCATTTTGGCTCAAG	G3183-030	GCCAAGGCCCACAAGTCAGCAT
	G3183-029	AACGCGGGCTTGCCTCAAGT	G2893-058	GAGTTGGGATGTGGTCAGCT
