# Supplementary material for: Transcriptomic characterization of the enzymatic antioxidants FeSOD, MnSOD, APX and KatG in the dinoflagellate genus Symbiodinium
Source: BMC Evol Biol. 2015 Mar 18;15:48. doi: 10.1186/s12862-015-0326-0 (PMC4416395; doi:10.1186/s12862-015-0326-0)
Supplement: Additional file 10: — Supplementary Methods. Detailed information on experimental setup, PAM fluorometry and growth measurements, sample processing and enzyme measurement for gene expression experiment. [file 12862_2015_326_MOESM10_ESM.pdf]

Supplementary Methods

Six batch cultures of *Symbiodinium* B1 (culture ID Ap1, obtained from the *Symbiodinium* culture collection, Santos Lab, Auburn University, Auburn, USA) were grown in 2 L round-bottom flasks containing silica-free sterile f/2-medium (pH 8.1) based on synthetic seawater (salinity 34; Instant Ocean Sea Salt, Spectrum Brands Inc., USA). Cultures were aerated with filter-sterilized air (0.22 µm) and grown on a constant 12-h/12-h light/dark cycle (cool white fluorescent tubes, Philips 36W/840) under a PAR irradiance of 40-50 µmol quanta m^{-2} s^{-1} (LI-COR Quantum light meter LI-189 with cosine sensor, LI-COR, Inc., USA) at 25°C in temperature-controlled tanks. After 12 days of acclimation under the experimental setting, cell aliquots from these six batch cultures served as inocula for 6 controls and 6 treatment flasks. Growth conditions after 12 days of acclimation were not nutrient-limited, and cultures were in log phase prior to starting the experiment. Initial densities were set to approximately 150,000 cells mL^{-1}. After sampling on Day 0, the temperature was increased to 33°C (1°C h^{-1}) in the treatment tank during the dark phase, while the control was maintained at 25°C. Based on the response in previous experiments, all cultures were sampled 6 hours after the beginning of the light phase on Days 0, 1, and 3 by taking a total volume of 350 mL (7 x 50 mL aliquots) on each sampling day. Total time for sampling was ca. 2 hours. Cells were pelleted (2000 x *g*, 5 min), flash frozen in liquid nitrogen and stored at -80°C. In addition, 5-10 mL aliquots were taken for determination of cell density (after fixation in 4 % formalin) and maximum quantum yield (F_v/F_m) via PAM fluorometry, both described below.

PAM fluorometry and growth measurements

After 20 min of dark acclimation, F_v/F_m of 2 mL of live culture was measured using a Water-PAM chlorophyll fluorometer (Heinz Walz GmbH, Effeltrich, Germany). Haemocytometer counts (N = 6) were used to calculate the specific growth rate (µ) for each replicate via a linear regression fit of log-transformed cell densities from all three sampling days [1]. Chl *a* was extracted from a 50 mL aliquot over 48 h in 1-2 mL N, N-dimethylformamide at 4°C in the dark. After centrifugation (5 min, 3900 x *g*, 4°C), 3 x 200 µL of the supernatant (technical triplicates) were measured at 646.8 nm, 663.8 nm and 750 nm in 96-well plates (UVStar, Greiner Bio-One GmbH, Frickenhausen, Germany). Chl *a* concentrations were determined after optical path length correction (0.555 cm) [2].

Sample processing

Three frozen pellets per replicate and time-point were pooled and lysed in cold buffer (50 mM KH_2PO_4/K_2HPO_4, 0.1 mM EDTA, 10% [v/v] glycerol) using ca. 200 mg glass beads (710-1180 µm; Sigma-Aldrich) in a bead mill (50 Hz, 3 min, 4°C; Qiagen tissue lyser, Qiagen N.V., Hilden, Germany). All lysates were centrifuged (16000 x *g*, 5 min, 4°C), and supernatants were aliquoted and frozen at -80°C until further analysis. Total aqueous soluble protein content was measured with the modified Bradford method using BSA as a protein standard [3].

Superoxide dismutase (SOD) assay

SOD assays were performed using the riboflavin/nitroblue tetrazolium (RF/NBT) assay in a microtiter plate format [4, 5]. Twenty microliters of lysate or SOD standard (0.5-500 U mL^{-1}) were measured as technical triplicates in a final reaction mixture of 300 µL potassium phosphate buffer (50 mM, pH 7.8) containing EDTA (0.1 mM), riboflavin (1.3 µM), L-methionine (10 mM), NBT (57 µM), and Triton X-100 (0.025% (v/v)). Absorbance was read at 560 nm both immediately and after 10 min incubation under a homogenous light field (130 µmol quanta m^{-2} s^{-1}) at 25°C. Standards and samples were measured using the same reaction mixture, and a sigmoidal, 5-parameter, semi logarithmic standard curve (24 standard levels) was used to infer the SOD activity of the samples. One unit of SOD activity was defined as the amount of enzyme that inhibits the reduction of NBT by 50%.

Ascorbate peroxidase (APX) assay

APX activity was assessed by monitoring the oxidation of ascorbate at 290 nm over 3 min at 25°C, using 100 µL lysate in a final reaction mixture of 700 µL potassium phosphate buffer (50 mM, pH 7.0), EDTA (0.1 mM), ascorbate (0.3 mM) and hydrogen peroxide (0.1 mM) [6] using a temperature-controlled cuvette spectrophotometer (UV-Vis Spectrophotometer UV-2550, Shimadzu Corp. Kyoto, Japan). APX activity was determined with $\varepsilon = 2.8$ mM^{-1} cm^{-1}.

Catalase peroxidase (KatG) assay

Catalase peroxidase activity was determined spectrophotometrically using 100 µL lysate in a total reaction volume of 700 µL, containing potassium phosphate buffer (50 mM, pH 7.0), EDTA (0.1 mM) and hydrogen peroxide (14 mM). The reaction was monitored for 3 min at 240 nm and 25°C in quartz cuvettes (UV-Vis Spectrophotometer UV-2550) and activities calculated using an extinction coefficient

of 43.6 M^{-1} cm^{-1} [7]. All enzyme activities were normalized per cell and expressed as specific activity (U $cell^{-1}$), where one unit catalyses one µmol substrate min^{-1} $cell^{-1}$.

References

1. Guillard RRL: **Division rates**. In: *Handbook of Phycological Methods - Culture Methods and Growth Measurements.* Edited by Stein JR. New York: Cambridge University Press; 1973: 298-311.
2. Porra R, Thompson W, Kriedemann P: **Determination of accurate extinction coefficients and simultaneous equations for assaying chlorophylls a and b extracted with four different solvents: verification of the concentration of chlorophyll standards by atomic absorption spectroscopy**. *Biochim Biophys Acta* 1989, **975**(3):384-394.
3. Zor T, Selinger Z: **Linearization of the Bradford protein assay increases its sensitivity: theoretical and experimental studies**. *Anal Biochem* 1996, **236**(2):302-308.
4. Beauchamp C, Fridovich I: **Superoxide dismutase: Improved assays and an assay applicable to acrylamide gels**. *Anal Biochem* 1971, **44**(1):276-287.
5. Fryer MJ, Andrews JR, Oxborough K, Blowers DA, Baker NR: **Relationship between CO_2 assimilation, photosynthetic electron transport, and active O_2 metabolism in leaves of maize in the field during periods of low temperature**. *Plant Physiol* 1998, **116**(2):571-580.
6. Nakano Y, Asada K: **Hydrogen peroxide is scavenged by ascorbate-specific peroxidase in spinach chloroplasts**. *Plant Cell Physiol* 1981, **22**(5):867-880.
7. Beers R, Sizer IW: **A spectrophotometric method for measuring the breakdown of hydrogen peroxide by catalase**. *J Biol Chem* 1952, **195**(1):133-140.
