# Supplementary material for: Transcriptomic characterization of the enzymatic antioxidants FeSOD, MnSOD, APX and KatG in the dinoflagellate genus Symbiodinium
Source: BMC Evol Biol. 2015 Mar 18;15:48. doi: 10.1186/s12862-015-0326-0 (PMC4416395; doi:10.1186/s12862-015-0326-0)
Supplement: Additional file 11: — Sequence details. List of biogeographic origin and sequence IDs and accession numbers of used contigs for all antioxidant sequences presented in this study. New sequences generated in this study are highlighted (*). [file 12862_2015_326_MOESM11_ESM.pdf]

Table S2. List of biogeographic origin and sequence IDs and accession numbers of used contigs for all antioxidant sequences presented in this study. New sequences generated in this study are highlighted (*).

**Gene**	**Sequence name**	**ITS2 type**	**Culture ID**	**Host**	**Geographic origin**	**Contigs used for sequence assembly**
MnSOD	A1 Casskb8 MnSOD _c18645	A1	Casskb8	*Cassiopea xamachana*	Hawai'i	kb8_c18645
MnSOD	A1 Casskb8 MnSOD rep_c710	A1	Casskb8	*Cassiopea xamachana*	Hawai'i	kb8_rep_c710
MnSOD	A1 Casskb8 SymMnSOD3 rep_c4192	A1	Casskb8	*Cassiopea xamachana*	Hawai'i	kb8_rep_c4192
MnSOD	A1 CCMP2467 MnSOD Assembly1	A1	CCMP2467	*Stylophora pistillata*	Red Sea	gi|556381675|gb|GAKY01035952.1| gi|556381673|gb|GAKY01035954.1| gi|556381670|gb|GAKY01035956.1| gi|556381668|gb|GAKY01035958.1| gi|556375787|gb|GAKY01041471.1|
MnSOD	A1 CCMP2467 MnSOD Assembly3	A1	CCMP2467	*Stylophora pistillata*	Red Sea	gi|556374884|gb|GAKY01042229.1| gi|556374883|gb|GAKY01042230.1| gi|556374881|gb|GAKY01042231.1| gi|556374878|gb|GAKY01042234.1|
MnSOD	A1 CCMP2467 MnSOD Assembly4	A1	CCMP2467	*Stylophora pistillata*	Red Sea	gi|556374880|gb|GAKY01042232.1| gi|556374879|gb|GAKY01042233.1| gi|556242634|gb|GAKY01158064.1|
MnSOD	A1 CCMP2467 SymMnSOD3 Assembly2	A1	CCMP2467	*Stylophora pistillata*	Red Sea	gi|556202041|gb|GAKY01194558.1| gi|556202040|gb|GAKY01194559.1| gi|556202039|gb|GAKY01194560.1| gi|556202038|gb|GAKY01194561.1| gi|556202037|gb|GAKY01194562.1|
MnSOD	A1 CCMP2467 SymMnSOD3 Assembly6	A1	CCMP2467	*Stylophora pistillata*	Red Sea	gi|556239202|gb|GAKY01161046.1| gi|556202036|gb|GAKY01194563.1|

*MnSOD	B1 Ap1 SymMnSOD1 KJ672521	B1	Ap1	*Aiptasia pulchella*	Hawai'i	GenBank KJ672521
MnSOD	B1 Mf1.05b MnSOD _c29099	B1	Mf1.05b	*Orbicella faveolata*	Florida Keys	mf105_c29099
MnSOD	B1 Mf1.05b SymMnSOD1 Assembly2	B1	Mf1.05b	*Orbicella faveolata*	Florida Keys	mf105_rep_c3255<br>mf105_s63926<br>mf105_s64469
MnSOD	B1 Mf1.05b SymMnSOD2 Assembly1	B1	Mf1.05b	*Orbicella faveolata*	Florida Keys	mf105_c50851<br>mf105_rep_c50851<br>mf105_rep_c9010<br>mf105_s74052
MnSOD	B1 Mf1.05b SymMnSOD2 rep_c13368	B1	Mf1.05b	*Orbicella faveolata*	Florida Keys	mf105_rep_c13368
MnSOD	B1 Mf1.05b SymMnSOD3 _c19510	B1	Mf1.05b	*Orbicella faveolata*	Florida Keys	mf105_c19510
MnSOD	C1 CCMP2466 SymMnSOD1 AHI54358	C1	CCMP2466	*Discosoma sanctithomae*	Jamaica	GenBank AHI54358
*MnSOD	C15 M.digitata SymMnSOD1 KJ672522	C15	N/A	*Montipora digitata*	Great Barrier Reef	GenBank KJ672522
MnSOD	C3 A.aspera SymMnSOD1 FE866047	C3	N/A	*Acropora aspera*	Great Barrier Reef	GenBank FE866047
*MnSOD	C3 Mp SymMnSOD1 KJ672520	C3	Mp	*Mastigias papua*	Palau	GenBank KJ672520
MnSOD	D A.hyacinthus SymMnSOD2 GAFP01017905	D	N/A	*Acropora hyacinthus*	American Samoa	gi|452163680|gb|GAFP01017905.1|
MnSOD	D A.hyacinthus SymMnSOD3 GAFP01006955	D	N/A	*Acropora hyacinthus*	American Samoa	gi|452174630|gb|GAFP01006955.1|
MnSOD	F1 CCMP2468 SymMnSOD2 Assembly1	F1	CCMP2468	*Montipora capitata*	Hawai'i	gi|512780496|gb|KC937118.1| gi|512783950|gb|KC940572.1| gi|512788616|gb|KC945238.1| gi|512788962|gb|KC945584.1| gi|512793081|gb|KC949703.1| gi|512793147|gb|KC949769.1| gi|512794392|gb|KC951014.1|
FeSOD	A PF-2005 SymFeSOD AY916504	A	PF-2005	?	?	GenBank AY916504
*FeSOD	B1 Ap1 SymFeSOD KJ672519	B1	Ap1	*Aiptasia pulchella*	Hawai'i	GenBank KJ672519

*FeSOD	E CCMP421 SymFeSOD KJ672517	E	CCMP421	free-living	New Zealand	GenBank KJ672517
*FeSOD	F1 Mv SymFeSOD KJ672518	F1	Mv	*Montipora capitata*	Hawai'i	GenBank KJ672518
APX	A1 Casskb8 APX Assembly4	A1	Casskb8	*Cassiopea xamachana*	Hawai'i	kb8_rep_c17856<br>kb8_rep_c21314
APX	A1 Casskb8 SymAPX rep_c1337	A1	Casskb8	*Cassiopea xamachana*	Hawai'i	kb8_rep_c1337
APX	A1 Casskb8 SymAPX1 rep_c1302	A1	Casskb8	*Cassiopea xamachana*	Hawai'i	kb8_rep_c1302
APX	A1 Casskb8 SymAPX1 rep_c1797	A1	Casskb8	*Cassiopea xamachana*	Hawai'i	kb8_rep_c1797
APX	A1 Casskb8 SymAPX2 Assembly1	A1	Casskb8	*Cassiopea xamachana*	Hawai'i	kb8_rep_c25023<br>kb8_rep_c47803<br>kb8_rep_c53272<br>kb8_s59539<br>kb8_s66605
APX	A1 Casskb8 SymAPX3 Assembly2	A1	Casskb8	*Cassiopea xamachana*	Hawai'i	kb8_rep_c161<br>kb8_s59940<br>kb8_s66277
APX	A1 Casskb8 SymAPX3 Assembly3	A1	Casskb8	*Cassiopea xamachana*	Hawai'i	kb8_rep_c2735<br>kb8_s66202
APX	A1 Casskb8 SymAPX3 rep_c280	A1	Casskb8	*Cassiopea xamachana*	Hawai'i	kb8_rep_c280
APX	A1 Casskb8 SymAPX5 rep_c18778	A1	Casskb8	*Cassiopea xamachana*	Hawai'i	kb8_rep_c18778
APX	A1 CCMP2467 APX Locus_35678	A1	CCMP2467	*Stylophora pistillata*	Red Sea	gi|556278914|gb|GAKY01124934.1|
APX	A1 CCMP2467 SymAPX1 Assembly8	A1	CCMP2467	*Stylophora pistillata*	Red Sea	gi|556203728|gb|GAKY01192980.1|+ Locus_33092
APX	A1 CCMP2467 SymAPX1 GAKY01045714	A1	CCMP2467	*Stylophora pistillata*	Red Sea	gi|556370780|gb|GAKY01045714.1|
APX	A1 CCMP2467 SymAPX1 Locus_51655	A1	CCMP2467	*Stylophora pistillata*	Red Sea	gi|556237089|gb|GAKY01162872.1|

						gi|556237087|gb|GAKY01162873.1| gi|556237086|gb|GAKY01162874.1|
APX	A1 CCMP2467 SymAPX1 Locus_9887	A1	CCMP2467	*Stylophora pistillata*	Red Sea	gi|556370789|gb|GAKY01045707.1| gi|556370788|gb|GAKY01045708.1| gi|556370779|gb|GAKY01045715.1|
*APX	A1 CCMP2467 SymAPX2 KJ672516	A1	CCMP2467	*Stylophora pistillata*	Red Sea	NCBI KJ672516
APX	A1 CCMP2467 SymAPX2 Locus_1984	A1	CCMP2467	*Stylophora pistillata*	Red Sea	gi|556411510|gb|GAKY01009605.1| gi|556411509|gb|GAKY01009606.1| gi|556411508|gb|GAKY01009607.1| gi|556411507|gb|GAKY01009608.1| gi|556411506|gb|GAKY01009609.1| gi|556411504|gb|GAKY01009610.1|
APX	A1 CCMP2467 SymAPX3 Assembly4	A1	CCMP2467	*Stylophora pistillata*	Red Sea	gi|556282844|gb|GAKY01121695.1| gi|556248744|gb|GAKY01152490.1| gi|556210679|gb|GAKY01186456.1| gi|556210677|gb|GAKY01186457.1|
APX	A1 CCMP2467 SymAPX3 Locus_31699	A1	CCMP2467	*Stylophora pistillata*	Red Sea	gi|556288848|gb|GAKY01116859.1| gi|556288847|gb|GAKY01116860.1| gi|556288845|gb|GAKY01116861.1| gi|556288844|gb|GAKY01116862.1| gi|556288843|gb|GAKY01116863.1| gi|556288842|gb|GAKY01116864.1|
APX	A1 CCMP2467 SymAPX5 Locus_10740	A1	CCMP2467	*Stylophora pistillata*	Red Sea	gi|556366894|gb|GAKY01049344.1| gi|556366892|gb|GAKY01049346.1| gi|556366889|gb|GAKY01049349.1|
APX	A1 CCMP2467 SymAPX6 Locus_36434	A1	CCMP2467	*Stylophora pistillata*	Red Sea	gi|556275911|gb|GAKY01127507.1| gi|556275910|gb|GAKY01127508.1|
*APX	B1 Ap1 SymAPX1 KJ672513	B1	Ap1	*Aiptasia pulchella*	Hawai'i	GenBank KJ672513

APX	B1 Mf1.05b SymAPX1 Assembly1	B1	Mf1.05b	*Orbicella faveolata*	Florida Keys	mf105_rep_c1375<br>mf105_rep_c20868
APX	B1 Mf1.05b SymAPX1 Assembly3	B1	Mf1.05b	*Orbicella faveolata*	Florida Keys	mf105_rep_c1624<br>mf105_s62425
APX	B1 Mf1.05b SymAPX1 Assembly4	B1	Mf1.05b	*Orbicella faveolata*	Florida Keys	mf105_c30166<br>mf105_s54996
APX	B1 Mf1.05b SymAPX1 rep_c30083	B1	Mf1.05b	*Orbicella faveolata*	Florida Keys	mf105_rep_c30083
APX	B1 Mf1.05b SymAPX1 rep_c960	B1	Mf1.05b	*Orbicella faveolata*	Florida Keys	mf105_rep_c960
APX	B1 Mf1.05b SymAPX2 rep_c23432	B1	Mf1.05b	*Orbicella faveolata*	Florida Keys	mf105_rep_c23432
APX	B1 Mf1.05b SymAPX4 Assembly2	B1	Mf1.05b	*Orbicella faveolata*	Florida Keys	mf105_rep_c287<br>mf105_s64607<br>mf105_s71948
APX	B1 Mf1.05b SymAPX6 rep_c6573	B1	Mf1.05b	*Orbicella faveolata*	Florida Keys	mf105_rep_c6573
*APX	C1 CCMP2466 SymAPX1 KF835562	C1	CCMP2466	*Discosoma sanctithomae*	Jamaica	GenBank KF835562
*APX	C15 M.digitata SymAPX1 KJ672515	C15	N/A	*Montipora digitata*	Great Barrier Reef	GenBank KJ672515
APX	C3 A.aspera SymAPX1 FE865713	C3	N/A	*Acropora aspera*	Great Barrier Reef	gi|186958871|gb|FE865713.1|FE865713
APX	C3 A.aspera SymAPX2 Assembly1	C3	N/A	*Acropora aspera*	Great Barrier Reef	gi|169299971|gb|FE538076.1|FE538076<br>gi|169299972|gb|FE538077.1|FE538077
APX	C3 S.hystrix SymAPX1 HM156698	C3	N/A	*Seriatopora hystrix*	Republic of China	NCBI HM156698
APX	D A.hyacinthus SymAPX1 GAFP01005798	D	N/A	*Acropora hyacinthus*	American Samoa	gi|452175787|gb|GAFP01005798.1|
APX	D A.hyacinthus SymAPX1 GAFP01021256	D	N/A	*Acropora hyacinthus*	American Samoa	gi|452160329|gb|GAFP01021256.1|
APX	D A.hyacinthus SymAPX2 GAFP01007188	D	N/A	*Acropora hyacinthus*	American Samoa	gi|452174397|gb|GAFP01007188.1|
APX	D A.hyacinthus SymAPX2 GAFP01018157	D	N/A	*Acropora hyacinthus*	American Samoa	gi|452163428|gb|GAFP01018157.1|
APX	D A.hyacinthus SymAPX3 GAFP01006424	D	N/A	*Acropora hyacinthus*	American Samoa	gi|452175161|gb|GAFP01006424.1|
APX	D A.hyacinthus SymAPX4 GAFP01007747	D	N/A	*Acropora hyacinthus*	American Samoa	gi|452173838|gb|GAFP01007747.1|
APX	D A.hyacinthus SymAPX5 Assembly1	D	N/A	*Acropora hyacinthus*	American Samoa	gi|452178808|gb|GAFP01002806.1|

						gi|452181480|gb|GAFP01000134.1|
APX	F1 CCMP2468 SymAPX1 Assembly2	F1	CCMP2468	*Montipora capitata*	Hawai'i	gi|512790905|gb|KC947527.1| gi|512792839|gb|KC949461.1| gi|512793621|gb|KC950243.1| gi|512794239|gb|KC950861.1|
APX	F1 CCMP2468 SymAPX1 Assembly3	F1	CCMP2468	*Montipora capitata*	Hawai'i	gi|512786816|gb|KC943438.1| gi|512791389|gb|KC948011.1| gi|512792900|gb|KC949522.1| gi|512793754|gb|KC950376.1|
APX	F1 CCMP2468 SymAPX1 Assembly7	F1	CCMP2468	*Montipora capitata*	Hawai'i	gi|512787724|gb|KC944346.1| gi|512794427|gb|KC951049.1|
APX	F1 CCMP2468 SymAPX4 KC944388	F1	CCMP2468	*Montipora capitata*	Hawai'i	gi|512787766|gb|KC944388.1|
*APX	F1 Mv SymAPX1 KJ672514	F1	Mv	*Montipora capitata*	Hawai'i	GenBank KJ672514
KatG	A1 Casskb8 SymKatG1 Assembly2	A1	Casskb8	*Cassiopea xamachana*	Hawai'i	kb8_c15217<br>kb8_c40578<br>kb8_rep_c52653<br>kb8_rep_c52669
*KatG	A1 CCMP2467 SymKatG1 KJ735681	A1	CCMP2467	*Stylophora pistillata*	Red Sea	GenBank KJ735681
KatG	A1 CCMP2467 SymKatG1 Assembly1	A1	CCMP2467	*Stylophora pistillata*	Red Sea	gi|556313331|gb|GAKY01096276.1| gi|556313326|gb|GAKY01096280.1| gi|556313330|gb|GAKY01096277.1| gi|556313328|gb|GAKY01096279.1| gi|556313329|gb|GAKY01096278.1|
KatG	A1 CCMP2467 SymKatG1 Assembly3	A1	CCMP2467	*Stylophora pistillata*	Red Sea	gi|556419849|gb|GAKY01002437.1| gi|556419848|gb|GAKY01002438.1| gi|556419847|gb|GAKY01002439.1|
*KatG	B1 Ap1 SymKatG1 KJ672511	B1	Ap1	*Aiptasia pulchella*	Hawai'i	GenBank KJ672511
KatG	B1 Mf1.05b SymKatG1 Assembly1	B1	Mf1.05b	*Orbicella faveolata*	Florida Keys	mf105_rep_c11051<br>mf105_rep_c14326

						mf105_rep_c169<br>mf105_rep_c35625<br>mf105_rep_c50341<br>mf105_s50647<br>mf105_s58339<br>mf105_s62531<br>mf105_s72023<br>mf105_s74129
*KatG	C1 CCMP2466 SymKatG1 KF835563	C1	CCMP2466	*Discosoma sanctithomae*	Jamaica	GenBank KF835563
KatG	C3 A.aspera SymKatG1 Assembly1	C3	N/A	*Acropora aspera*	Great Barrier Reef	gi|145309691|gb|EH036045.1|EH036045<br>gi|145309692|gb|EH036046.1|EH036046<br>gi|145309693|gb|EH036047.1|EH036047<br>gi|145309694|gb|EH036048.1|EH036048<br>gi|186961140|gb|FE866054.1|FE866054
KatG	D A.hyacinthus SymKatG1 GAFP01010732	D	N/A	*Acropora hyacinthus*	American Samoa	gi|452170853|gb|GAFP01010732.1|
KatG	D A.hyacinthus SymKatG1 GAFP1022639	D	N/A	*Acropora hyacinthus*	American Samoa	gi|452158946|gb|GAFP01022639.1|
*KatG	E CCMP421 SymKatG1 KJ672510	E	CCMP421	free-living	New Zealand	GenBank KJ672510
*KatG	F1 Mv SymKatG1 KJ672509	F1	Mv	*Montipora capitata*	Hawai'i	GenBank KJ672509
KatG	A1 Casskb8 SymKatG2 _c15386	A1	Casskb8	*Cassiopea xamachana*	Hawai'i	kb8_c15386
KatG	A1 CCMP2467 SymKatG2 Assembly2	A1	CCMP2467	*Stylophora pistillata*	Red Sea	gi|556242872|gb|GAKY01157826.1| gi|556242873|gb|GAKY01157825.1| gi|556374816|gb|GAKY01042285.1| gi|556374818|gb|GAKY01042284.1|
KatG	B1 Mf1.05b SymKatG2 c_32035	B1	Mf1.05b	*Orbicella faveolata*	Florida Keys	mf105_c32035
KatG	D A.hyacinthus SymKatG2 GAFP01010883	D	N/A	*Acropora hyacinthus*	American Samoa	gi|452170702|gb|GAFP01010883.1|
