# Supplementary material for: Transcriptomic characterization of the enzymatic antioxidants FeSOD, MnSOD, APX and KatG in the dinoflagellate genus Symbiodinium
Source: BMC Evol Biol. 2015 Mar 18;15:48. doi: 10.1186/s12862-015-0326-0 (PMC4416395; doi:10.1186/s12862-015-0326-0)
Supplement: Additional file 7: — KatG pairwise amino acid identities. Pairwise amino acid sequence identities of KatG sequences from different Symbiodinium ITS2 types based on the alignment in Figure 5. Pairwise identities between full length sequences (green) and pairwise identities within clade C (yellow) have been highlighted. [file 12862_2015_326_MOESM7_ESM.pdf]

		Full length	Ungapped length [aa]	A1 Casskb8 SymKatG1 Assembly2	A1 CCMP2467 SymKatG1 KJ735681	A1 CCMP2467 SymKatG1 Assembly3	A1 CCMP2467 SymKatG1 Assembly1	B1 Ap1 SymKatG1 KJ672511	B1 Mf1.05b SymKatG1 Assembly1	C1 CCMP2466 SymKatG1 KF835563	C3 A.aspera SymKatG1 Assembly1	D A.hyacinthus SymKatG1 GAFP1022639	D A.hyacinthus SymKatG1 GAFP01010732	E CCMP421 SymKatG1 KJ672510	F1 Mv SymKatG1 KJ672509	A1 Casskb8 SymKatG2 _c15386	A1 CCMP2467 SymKatG2 Assembly2	B1 Mf1.05b SymKatG2 c_32035	D A.hyacinthus SymKatG2 GAFP01010883
				1	2	3	4	5	6	7	8	9	10	11	12	13	14	15	16
		Full length							Y										Y
			Ungapped length [aa]	355	230	93	83	363	486	397	370	216	143	335	289	364	368	231	423
A1 Casskb8 SymKatG1 Assembly2	1		355		100%	99%	N/A	75%	72%	78%	77%	74%	100%	77%	79%	39%	39%	36%	36%
A1 CCMP2467 SymKatG1 KJ735681	2		230	100%		100%	N/A	71%	69%	78%	79%	74%	N/A	76%	79%	39%	39%	35%	35%
A1 CCMP2467 SymKatG1 Assembly3	3		93	99%	100%		N/A	75%	75%	83%	82%	80%	N/A	83%	84%	42%	42%	42%	40%
A1 CCMP2467 SymKatG1 Assembly1	4		83	N/A	N/A	N/A		69%	69%	66%	66%	N/A	76%	87%	67%	39%	39%	N/A	36%
B1 Ap1 SymKatG1 KJ672511	5		363	75%	71%	75%	69%		100%	84%	83%	75%	67%	87%	87%	34%	34%	40%	35%
B1 Mf1.05b SymKatG1 Assembly1	6	Y	486	72%	69%	75%	69%	100%		84%	83%	73%	67%	86%	87%	36%	36%	34%	34%
C1 CCMP2466 SymKatG1 KF835563	7		397	78%	78%	83%	66%	84%	84%		98%	80%	68%	90%	92%	35%	35%	40%	37%
C3 A.aspera SymKatG1 Assembly1	8		370	77%	79%	82%	66%	83%	83%	98%		79%	70%	90%	91%	36%	36%	39%	36%
D A.hyacinthus SymKatG1 GAFP1022639	9		216	74%	74%	80%	N/A	75%	73%	80%	79%		N/A	79%	80%	39%	40%	34%	35%
D A.hyacinthus SymKatG1 GAFP01010732	10		143	100%	N/A	N/A	76%	67%	67%	68%	70%	N/A		90%	83%	33%	33%	N/A	33%
E CCMP421 SymKatG1 KJ672510	11		335	77%	76%	83%	87%	87%	86%	90%	90%	79%	90%		90%	40%	40%	39%	39%
F1 Mv SymKatG1 KJ672509	12		289	79%	79%	84%	67%	87%	87%	92%	91%	80%	83%	90%		40%	40%	40%	40%
A1 Casskb8 SymKatG2 _c15386	13		364	39%	39%	42%	39%	34%	36%	35%	36%	39%	33%	40%	40%		100%	77%	76%
A1 CCMP2467 SymKatG2 Assembly2	14		368	39%	39%	42%	39%	34%	36%	35%	36%	40%	33%	40%	40%	100%		76%	76%
B1 Mf1.05b SymKatG2 c_32035	15		231	36%	35%	42%	N/A	40%	34%	40%	39%	34%	N/A	39%	40%	77%	76%		81%
D A.hyacinthus SymKatG2 GAFP01010883	16	Y	423	36%	35%	40%	36%	35%	34%	37%	36%	35%	33%	39%	40%	76%	76%	81%	
